# *Phytophthora infestans* RXLR effector AVR1 disturbs the growth of *Physcomitrium patens* without affecting Sec5 localization

**DOI:** 10.1371/journal.pone.0249637

**Published:** 2021-04-08

**Authors:** Elysa J. R. Overdijk, Vera Putker, Joep Smits, Han Tang, Klaas Bouwmeester, Francine Govers, Tijs Ketelaar

**Affiliations:** 1 Laboratory of Cell Biology, Wageningen University & Research, Wageningen, The Netherlands; 2 Laboratory of Phytopathology, Wageningen University & Research, Wageningen, The Netherlands; 3 Biosystematics Group, Wageningen University & Research, Wageningen, The Netherlands; University of Nebraska-Lincoln, UNITED STATES

## Abstract

Plant pathogens often exploit a whole range of effectors to facilitate infection. The RXLR effector AVR1 produced by the oomycete plant pathogen *Phytophthora infestans* suppresses host defense by targeting Sec5. Sec5 is a subunit of the exocyst, a protein complex that is important for mediating polarized exocytosis during plant development and defense against pathogens. The mechanism by which AVR1 manipulates Sec5 functioning is unknown. In this study, we analyzed the effect of AVR1 on Sec5 localization and functioning in the moss *Physcomitrium patens*. *P*. *patens* has four Sec5 homologs. Two (PpSec5b and PpSec5d) were found to interact with AVR1 in yeast-two-hybrid assays while none of the four showed a positive interaction with AVR1^ΔT^, a truncated version of AVR1. In *P*. *patens* lines carrying β-estradiol inducible *AVR1* or *AVR1*^*ΔT*^ transgenes, expression of *AVR1* or *AVR1*^*ΔT*^ caused defects in the development of caulonemal protonema cells and abnormal morphology of chloronema cells. Similar phenotypes were observed in *Sec5*- or *Sec6*-silenced *P*. *patens* lines, suggesting that both AVR1 and AVR1^ΔT^ affect exocyst functioning in *P*. *patens*. With respect to Sec5 localization we found no differences between β-estradiol-treated and untreated transgenic AVR1 lines. Sec5 localizes at the plasma membrane in growing caulonema cells, also during pathogen attack, and its subcellular localization is the same, with or without AVR1 in the vicinity.

## Introduction

*Phytophthora* species are oomycete plant pathogens that are widespread and cause substantial damage in agriculture, forestry and nature [1]. *Phytophthora infestans*, one of the most notorious species, causes late blight, a disease that leads to large yield losses in potato and tomato production worldwide. Most plant pathogens promote host colonization by secreting effectors that suppress plant defense. Genome sequencing revealed that *Phytophthora* species have a few hundred genes predicted to encode RXLR effectors [2, 3]. These effectors are named after a conserved motif consisting of the amino acids arginine—any amino acid–leucine—arginine (RXLR) and are secreted during the infection process [3–5]. The RXLR motif is located in the N-terminal part adjacent to the signal peptide and precedes a highly variable C-terminal region that determines effector activity and host specificity [6–9].

*Phytophthora* species exploit multiple strategies to suppress plant defense. The many RXLR effectors target a vast array of host proteins thereby manipulating the host cell machinery at different levels and subcellular locations. For several *P*. *infestans* RXLR effectors, host targets have been identified. The RXLR effector IPI-O disrupts adhesion between the plant plasma membrane (PM) and the cell wall, potentially via interacting with lectin receptor kinases [10]. AVR3a was shown to target and stabilize the E3 ubiquitin ligase CMPG1 to suppress plant immunity [11], whereas Pi03192 prevents re-localization of two plant NAC transcription factors to the nucleus thereby suppressing defense activation [12]. PexRD2 was shown to interact with the kinase domain of MAPKKKε leading to disruption of the MAPK defense signaling pathway [13]. Besides suppressing plant defense, several RXLR effectors are recognized by intracellular nucleotide-binding leucine-rich repeat (NLR) resistance (R) proteins, and hence called avirulence (AVR) proteins. This effector-triggered immunity results in a hypersensitive response (HR) manifested by localized cell death to restrict pathogen invasion [14]. This is for example the case for AVR3a that is encoded by two alleles that only differ in three amino acids. *P*. *infestans* isolates expressing the *Avr3a* allele encoding AVR3a^KI^ are recognized by NLR R-protein R3a thereby triggering HR in potato cultivars carrying the *R3a* resistance gene, while isolates expressing the *avr3a* allele encoding AVR3a^EM^ escape recognition by R3a [6].

Similarly, RXLR effector AVR1 from *P*. *infestans* is recognized by the corresponding potato NLR R-protein R1 and this triggers a strong HR resulting in R1-mediated resistance [15]. *P*. *infestans* isolates able to colonize *R1*-potato plants were found to lack *AVR1* but do contain a homolog named *AVR1-like* (*A-L*) [16, 17]. A-L is highly similar to AVR1 (82% homology at the protein level) but shorter than AVR1 because it lacks the T-region, a tail of 38 amino acids at the very end of the C-terminus. A-L is not recognized by R1 and therefore isolates carrying *A-L* instead of *AVR1* do not trigger R1-mediated HR [17].

During infection of a non-*R1* potato cultivar, AVR1 acts as a virulence factor: it promotes colonization and suppresses defense responses in the host including callose deposition [15]. It does so by targeting the potato protein StSec5 [15]. Sec5 is a subunit of the exocyst, an octameric protein complex mediating polarized exocytosis by tethering exocytotic vesicles to the plasma membrane [18]. In plants, the exocyst functions in several developmental and defense-related processes, including cytokinesis, polarized tip growth and callose deposition [19–23]. Coimmunoprecipitation assays have shown that in plant cells (in this case in *Nicotiana benthamiana*) AVR1 directly interacts with StSec5. These assays also revealed that in the presence of AVR1, the amount of detectable StSec5 was increased, suggesting that AVR1 stabilizes StSec5 [15]. The finding that both *Sec5* silencing and transient expression of *AVR1* results in a decrease in callose deposition, raised the hypothesis that the AVR1-Sec5 interaction impairs exocyst function [15]. In contrast to AVR1, A-L and AVR1 lacking the T-region (AVR1^ΔT^) were not able to interact with StSec5, highlighting the importance of the T-region in host defense suppression [15].

Besides AVR1, a few other pathogen effectors were found to target exocyst subunits. One interaction that is confirmed by coimmunoprecipitation, is between AVR-Pii from the rice blast fungus *Magnaporthe oryzae* and rice Exo70, in particular Exo70F3 [24]. Other effectors that scored positive in yeast-two-hybrid (Y2H) screens as potentially interacting with *Arabidopsis* Sec5a, include the RXLR effectors RxL62 and RxL470 from the oomycete *Hyaloperonospora arabidopsidis* and the type III effector HopC1 from the bacterium *Pseudomonas syringae* [25], but as yet these interactions were not confirmed by other methods.

To visualize how the exocyst responds to pathogen attack and what happens at the cellular level when effectors target exocyst subunits, requires detailed imaging of the dynamics of the exocyst complex in living cells by high resolution microscopy. However, live-cell imaging of plant-pathogen interactions is often hampered by the tissue complexity and multi-cell layered nature of plants. In recent years there is an increased interest in studying defense responses in more distinctly related taxa, such as bryophytes. They have the advantage that they possess tissues that consist of a few, or even just one cell-layer and hence, are more accessible for high resolution microscopy [26]. Several bryophytes that initially emerged as models for plant developmental processes appear to be susceptible to a diverse range of pathogens and, similar to seed plants, show intimate relationships with beneficial microbes [27]. Most popular for host-microbe interaction studies are the moss *Physcomitrium patens* (formerly known as *Physcomitrella patens*) and the liverwort *Marchantia polymorpha*. Another attribute that makes these bryophytes popular model species is their amenability to genetic modification; because they are haploid it is feasible to replace endogenous genes by homologous recombination and generate lines producing fluorescently-tagged versions of proteins of interest.

In a previous study we showed that *P*. *patens* is a suitable host for *Phytophthora* pathogens [26]. *P*. *infestans* and *Phytophthora capsici*, a species with a broad host range, can both invade moss protonema cells and colonize the tissue. This is ideal for studying plant-*Phytophthora* interactions at the cellular level and is complementary to model systems with seed plants as hosts for *Phytophthora*. Live-cell microscopy of moss lines expressing LifeAct-GFP and mCherry-tagged tubulin, revealed cytoskeleton rearrangements in protonema cells shortly after inoculation with *P*. *capsici*, and in particular a rapid accumulation of actin filaments around the site of attack [26]. We also generated moss lines expressing GFP-tagged versions of three exocyst subunits, i.e. Sec3, Sec5 and Sec6 [28], and analysed their localization in protonema cells infected with *P*. *capsici* [29]. All three exocyst subunits appeared to accumulate at sites of attempted pathogen penetration and also on membranes surrounding papilla-structures and hyphal encasements in cells invaded by *P*. *capsici*. This re-localization suggests that the exocyst has a role in facilitating polarized exocytosis upon pathogen attack.

In this study we build on the finding that the *P*. *infestans* RXLR-effector AVR1 targets Sec5 and we exploit *P*. *patens* for unravelling mechanisms by which pathogen effectors potentially manipulate exocyst functioning. We first analyzed the Sec5 homologs in *P*. *patens* and selected two that interact with AVR1 in yeast-two-hybrid assays. We then expressed *AVR1* in *P*. *patens* lines with GFP-tagged Sec5 using a β-estradiol inducible expression system and investigated the effect of AVR1 on *P*. *patens* growth and Sec5 localization. We also generated *Sec5*-silenced *P*. *patens* lines and found a striking resemblance in phenotype between *Sec5*-silenced lines and lines expressing *AVR1*.

## Materials and methods

### Protein alignment and phylogenetic analysis

Protein sequence alignments were constructed using MAFFT and edited in Jalview. Phylogenetic trees were constructed using Phylogeny.fr and the percent identity matrix using Clustal Omega. Gene codes are listed in S1 Table in S1 File.

### Plasmids and cloning procedures

For yeast-two-hybrid expression constructs, coding sequences of *Sec5a*, *Sec5b*, *Sec5c* and *Sec5d* were PCR-amplified from a *P*. *patens* cDNA library (Gene IDs in S1 Table in S1 File). *Sec5c* was re-annotated and manually constructed by fusing the 616 bp upstream sequence obtained from genome assembly V1.6 to the *Sec5* coding sequence deduced from genome assembly V3.1 (retrieved from cosmoss.org). PCR products were cloned into the pENTR-D-TOPO vector and subsequently subcloned into destination plasmid pDEST22 (Invitrogen) via Gateway LR reactions. The construction of pDEST32 containing *AVR1*, *A-L* and *AVR1*^*ΔT*^ is described in a previous study [15]. The *4-myc-Cerulean-AVR1* and *4-myc-Cerulean-AVR1*^*ΔT*^ constructs were created by overlap PCR resulting in fragments that were subsequently introduced into the pENTR-D-TOPO vector and recombined into destination plasmid pDEST32 via Gateway LR reactions.

For β-estradiol inducible *AVR1* expression in *P*. *patens*, we used the system described in [30]. To generate a myc-tagged *AVR1*, we amplified *AVR1-10myc* from a pGWB20-AVR1-myc vector [15] and introduced this into the pENTR-D-TOPO vector. The same was done for *AVR1ΔT-10myc* after recombination of the pENTR-D-TOPO-AVR1^ΔT^ with pGWB20 (Invitrogen) via LR cloning. The pENTR-D-TOPO vectors containing *AVR1-myc*, *AVR1*^*ΔT*^*-myc*, *myc-Cer-AVR1* or *myc-Cer-AVR1*^*ΔT*^ were subsequently recombined into the β-estradiol inducible expression vector pPGX8 [30] via Gateway LR reactions (S2A Fig).

For β-estradiol inducible RNAi targeting of the four *PpSec5* transcripts, we used the system described in [31]. Four 450 base pair fragments of the coding sequence of *Sec5a*, *Sec5b*, *Sec5c* and *Sec5d* were amplified by PCR from a *P*. *patens* cDNA library and cloned into pENTR-D-TOPO and subsequently introduced into silencing vector pGG626 [31] via a Gateway LR reaction (S2A Fig). Primers and plasmids are listed in S2 and S3 Tables in S1 File, respectively.

#### Yeast-two-hybrid assays

Yeast-two-hybrid assays were performed with a split Gal4 transcription factor system using the *His3* gene as reporter [32]. PpSec5 proteins were fused with the Gal4 activation domain (AD: pDEST22) and AVR1 proteins with the binding domain (BD: pDEST32). The pDEST22/32-based constructs (S3 Table in S1 File) were transformed into yeast strain PJ69-4a or PJ69-4α by PEG-mediated transformation. All constructs were checked for autoactivation (S1 Fig). Yeast transformants with minimal background reporter activity were selected on double dropout medium (–Leu–His or–Trp–His) with varying concentrations of 3-amino-1,2,4-triazole (3-AT) to increase histidine-dependent growth stringency. Selected clones were mated and resulting diploids were selected on–Leu–Trp plates. With the surviving cells, a yeast-two-hybrid assay was performed on triple dropout medium (–Leu–Trp–His) with increasing concentrations of 3-AT.

### *P*. *patens* growth conditions and transformation

*P*. *patens* was grown on BCDAT plates under continuous light at 25°C [33]. Plasmids were linearized and introduced into the *P*. *patens* genome by homologous recombination using PEG-mediated protoplast transformation [33]. Insertion events were characterized by PCR (S2B Fig). The recipient *P*. *patens* strains used for transformation were the wildtype isolate Gransden [34], Sec5-GFP strains [28] and a mCherry-Tua1 strain (T. Miki, unpublished). The Sec6-silenced strain was generated previously as described in [28]. Moss lines used and generated in this study are listed in S4 Table in S1 File.

### *P*. *patens* phenotyping

For phenotyping at the colony level, *P*. *patens* protonemata of about 1 mm^2^ in size were grown on BCDAT medium covered with cellophane for two days. Subsequently, half of the replicas was transferred to BCDAT medium enriched with 1 μM β-estradiol to induce transgene expression, the other half was used as non-induced control. Growth of the colony was visualized at 0, 3 and 5 days after induction of expression by β-estradiol. *P*. *patens* cell morphology was visualized 3 days after culturing in special glass bottom dishes [35] containing BCD medium with and without β-estradiol.

### Quantitative RT-PCR

*P*. *patens* protonemal tissue was grown on BCDAT medium with and without β-estradiol for 2 days and total RNA was isolated using Trizol and a RNeasy mini kit (Qiagen). cDNA was synthesized on 1 μg of total RNA using a iScript cDNA synthesis kit (Bio-Rad). Quantitative RT-PCR was performed using a SYBR Hi-ROX kit (Bioline, London, UK), gene-specific primers (S2 Table in S1 File) and 3 μl of 10-times diluted cDNA using a Bio-Rad CFX96 Real-Time PCR system. Gene expression levels were normalized to *EF1α* transcript levels and to the transcript levels of the studied gene in non-induced moss tissue, according to the ΔΔCt method [36].

### Microscopy

Phenotyping was performed using a Nikon Hoffman modulation contrast microscope equipped with an Axiocam MRc5 with Axiovision software and 4 x (Leitz Wetzlar, NA 0.12) or 20 x (Nikon MC2, NA 0.4) objectives.

Fluorescent live-cell imaging was performed on a Roper spinning disk microscope system composed of a Nikon Ti eclipse body, Yokogawa CSU-X1 spinning disc head and a Photometrics Evolve 512 camera. Imaging was conducted with a 100x Plan Apo VC oil immersion objective (NA 1.40) using a 1.2x post-magnification fitted in front of the camera. GFP was excited using 491 nm light generated by a Cobolt Calypso50 laser, 30% power, and the emitted light was bandpass filtered at 497–560 nm. Cerulean was excited using a 405 nm Melles-Griot 56RCS001 laser, 85% power, and emission light was filtered through a 460/50 band pass filter. Typical exposures were 1000–2000 ms.

For live-cell imaging of infected cells we used time-gated confocal microscopy to eliminate undesired autofluorescence at infection sites [29]. *P*. *patens* was inoculated with *P*. *capsici* isolate LT263 and subsequently imaged as described in [29].

FIJI software [37] was used for all image analysis and processing.

## Results and discussion

### Interaction of AVR1 with two *P*. *patens* Sec5 homologs

**Fig 1:**
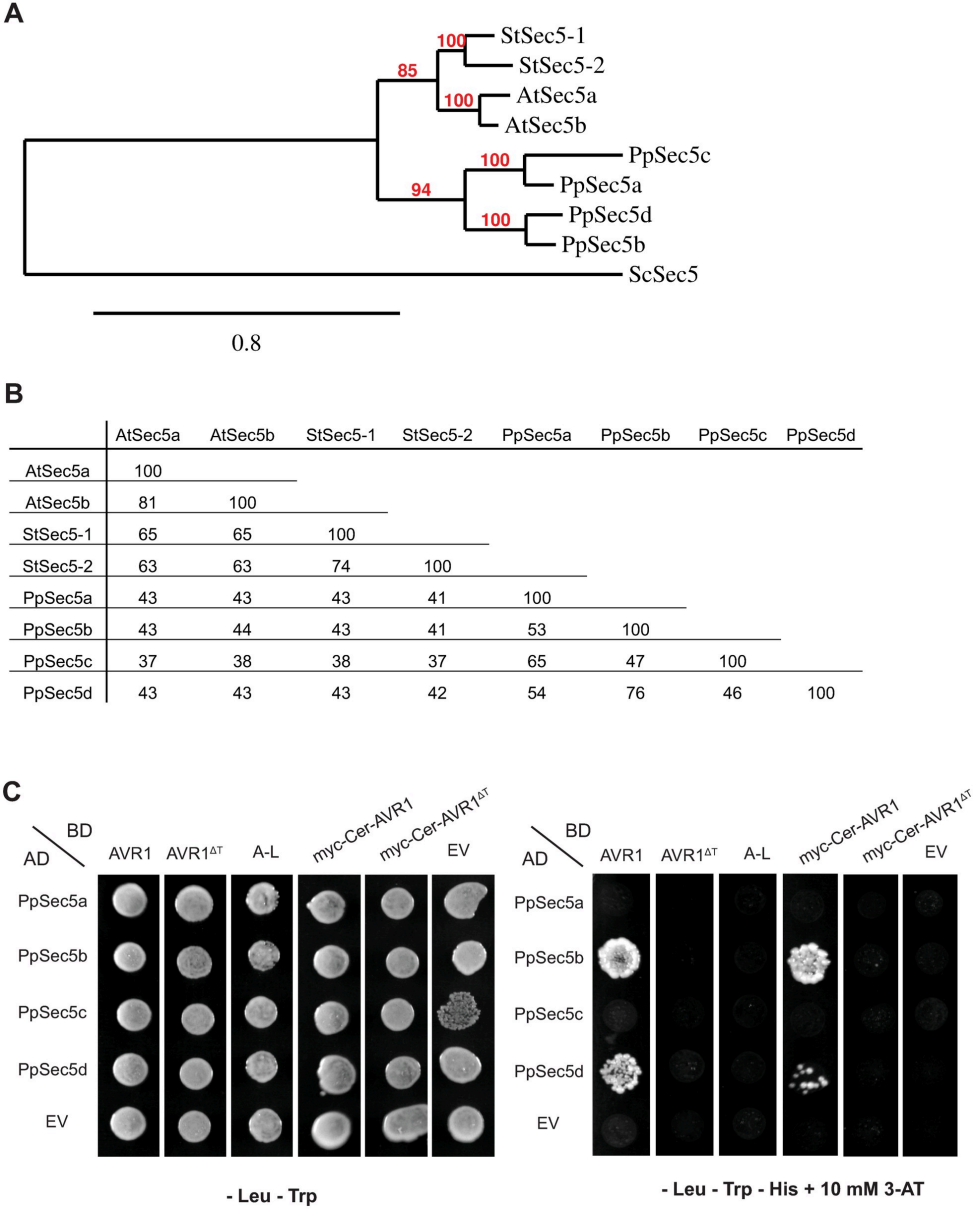
*Physcomitrium patens* has four Sec5 homologs with two showing interaction with *Phytophthora infestans* effector AVR1 in yeast. (A) Phylogenetic tree and (B) percent identity matrix of Sec5 proteins of *P*. *patens* (Pp), *Arabidopsis thaliana* (At) and potato (*Solanum tuberosum*, *St*). Gene codes are listed in S1 Table in S1 File. The phylogram in (A) was generated using the maximum likelihood method. Yeast (*Saccharomyces cerevisiae*, *Sc*) Sec5 served as an outgroup. Bootstrap values (red) indicate the percentage of trees in which the associated taxa cluster. Horizontal branch length represents the number of amino acid substitutions. (C) Yeast-two-hybrid assay to analyze the interaction between the four *P*. *patens Sec5* homologs and different versions of AVR1. Sec5 proteins were fused with the Gal4 activation domain (AD) and AVR 1 proteins with the binding domain (BD). Yeast strains co-expressing the different combinations were tested for successful mating on double dropout medium (-Leu-Trp) and for growth on triple dropout medium (–Leu–Trp–His) supplemented with 10 mM of 3-AT. EV: empty vector.

The *P*. *patens* genome contains four *Sec5* genes encoding proteins that share about 40% identity with Sec5 subunits of seed plants (Fig 1). To determine whether AVR1 interacts with *P*. *patens* Sec5 we performed yeast-two-hybrid (Y2H) assays, including control assays to exclude autoactivation (S1 Fig), and tested all four *P*. *patens* Sec5 homologs for their interaction with AVR1, AVR1^ΔT^ and A-L. The assays showed that AVR1 interacts with two Sec5 homologs, PpSec5b and PpSec5d, while AVR1^ΔT^ and A-L do not interact with any of the four *P*. *patens* Sec5 homologs (Fig 1C). The latter is in line with previous results showing that truncation of the T-region abolishes the interaction with potato StSec5 [15]. The binding to only two out of four Sec5 homologs might be due to the sequence divergence among the homologs. PpSec5b and PpSec5d have the highest pairwise similarity (76%) and group together in the phylogenetic tree, separated from the PpSec5a and PpSec5c (Fig 1A and 1B). Alignment of the four PpSec5 protein sequences with StSec5 revealed 41 amino acids that are identical in StSec5, PpSec5b and PpSec5d but different in PpSec5a and PpSec5c (S4 Fig). Whether or not these amino acids are determinants for the structure of Sec5 and/or the molecular interaction with AVR1 remains to be investigated.

### *AVR1* and *AVR1*^*ΔT*^ expression in *P*. *patens*

To study whether the presence of AVR1 inside plant cells affects the behavior of Sec5, we generated transgenic *P*. *patens* lines expressing *AVR1* or, as a control, *AVR1*^*ΔT*^. To avoid the risk of AVR1 causing developmental defects, thereby disturbing the transformation process, we used an inducible expression system mediated by β-estradiol [30]. We also included tags to facilitate detection of the proteins, i.e. a 10-myc-tag at the C-terminus or a 4-myc-tag combined with a fluorescent Cerulean-tag (Cer) at the N-terminus (S2A Fig). As shown previously the AVR1-myc fusion protein is still able to interact with StSec5 in *N*. *benthamiana* despite the presence of the tag [15]. Similarly, the myc-Cer-AVR1 fusion protein still interacts with PpSec5b and PpSec5d in the Y2H assay (Fig 1C), indicating that the N-terminal tag does not abolish the interaction of AVR1 with its target.

**Fig 2:**
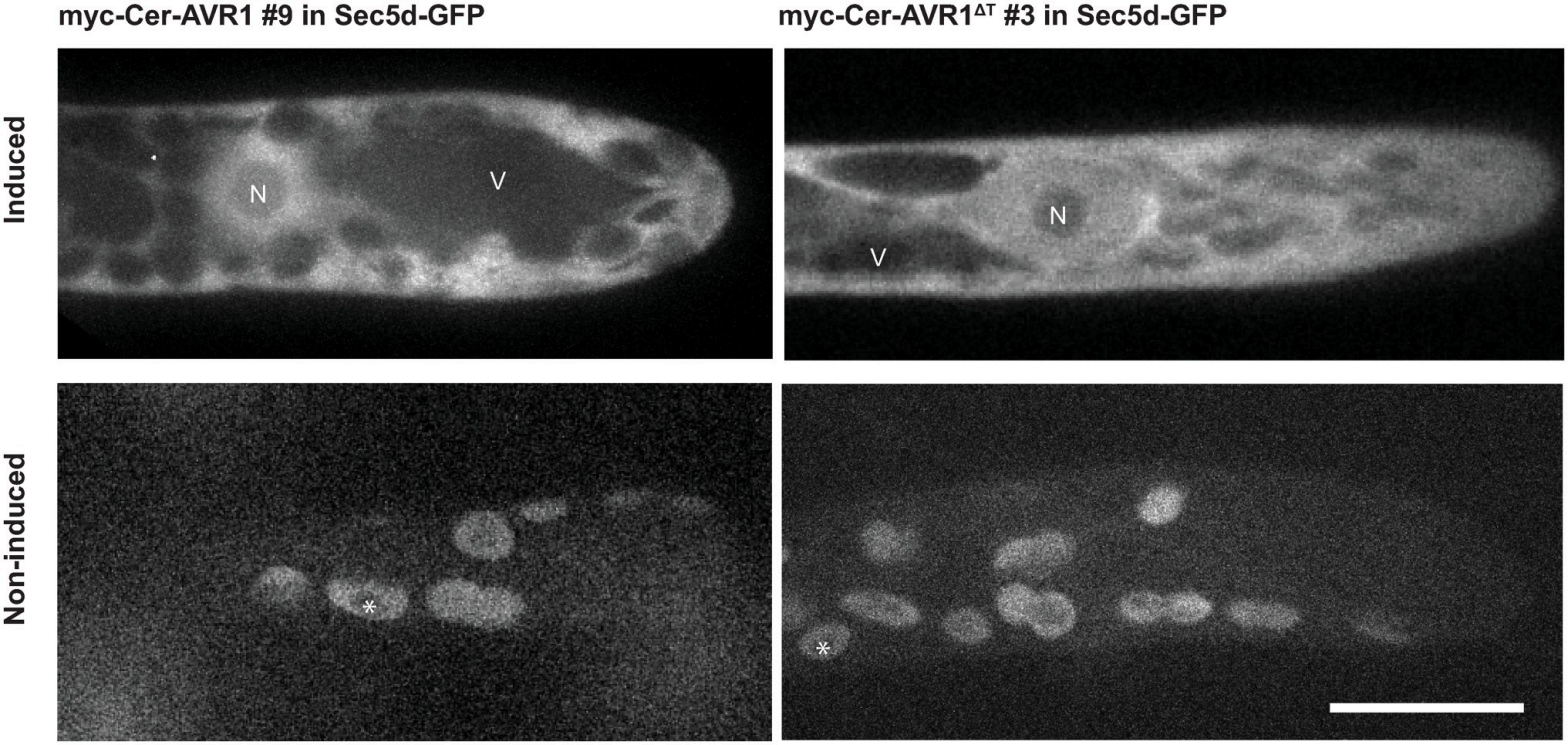
AVR1 and AVR1^ΔT^ localize in the cytoplasm and nucleus of *Physcomitrium patens*. Moss lines were grown for 3 days on BCD medium with or without β-estradiol (induced and non-induced, respectively) and examined by confocal fluorescence microscopy. Laser and filter settings were adjusted for excitation of cerulean. Images are maximum z-projections of 5 slides, 0.5 μm each (upper panels) or single confocal planes (lower panels). Scale bar represents 10 μm. N: nucleus, V: vacuole. Asterisks (lower panels) mark auto-fluorescence of chloroplasts.

The *AVR1* and *AVR1*^*ΔT*^ constructs were integrated via homologous recombination in two previously developed *P*. *patens* recipient lines; one containing *Sec5b-GFP* and the other *Sec5d-GFP* [28]. We selected about hundred stable transformants and verified the insertions of the four constructs by genotyping (S2B Fig). Successful induction of *AVR1* and *AVR1*^*ΔT*^ expression upon β-estradiol treatment was confirmed by confocal fluorescence microscopy (Fig 2). Myc-Cer-AVR1 and myc-Cer-AVR1^ΔT^ localized to the cytoplasm and nucleus of induced *P*. *patens* cells. Non-induced cells consistently showed chloroplast autofluorescence and no other fluorescence demonstrating that expression of *AVR1* and *AVR1*^*ΔT*^ is tightly regulated via β-estradiol induction. The nucleocytoplasmic localization of AVR1 in *P*. *patens* is in line with previous findings in *N*. *benthamiana* [16, 38] and characteristic for AVR1 function. Activation of R1-mediated immunity requires AVR1 localization in the nucleus, while in the absence of R1, host defense suppression is more effective when AVR1 is targeted to the cytoplasm [16].

### AVR1 and AVR1^ΔT^ affect growth and development of *P*. *patens* protonema cells

**Fig 3:**
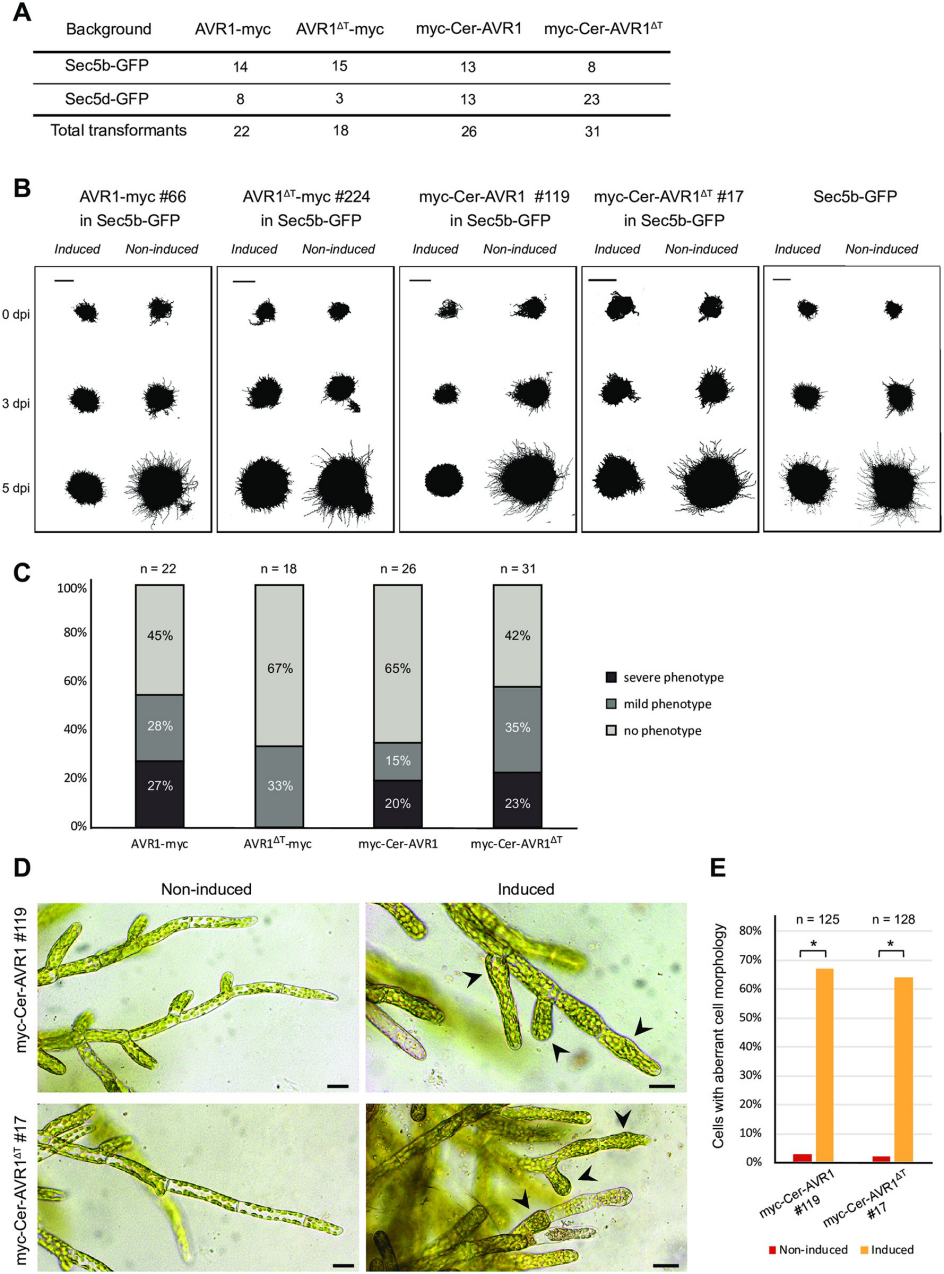
AVR1 and AVR1^ΔT^ reduce *Physcomitrium patens* growth. (A) Total number of stable transgenic *P*. *patens* lines containing inducible *AVR1* or *AVR1*^*ΔT*^. Four different constructs (*AVR1-myc*, *AVR1*^*ΔT*^*-myc*, *myc-Cer-AVR1* and *myc-Cer-AVR1*^*ΔT*^) were transformed into two background *P*. *patens* lines (Sec5b-GFP and Sec5d-GFP). (B) Growth phenotypes of *AVR1-* and *AVR1*^*ΔT*^-expressing *P*. *patens* lines. Moss lines were grown on BCDAT medium with (induced) and without (non-induced) β-estradiol. The images show colony growth of representative transformants at 0, 3 and 5 days after induction (dpi). This experiment was repeated three times with comparable results. Scale bars represent 2 mm. (C) Phenotype index comprising all *AVR1-* and *AVR1*^*ΔT*^-expressing transformants. Transformants were grown on BCDAT medium with and without β-estradiol for 7 days and moss colony phenotypes were scored by microscopy. The phenotype was scored as severe when there was no caulonema outgrowth from the moss colony (e.g. myc-Cer-AVR1 #119 imaged in (B)) and as mild when the caulonema outgrowth was at least 30% reduced compared to the non-induced colony (e.g. AVR1^ΔT^-myc #224 imaged in (B)). (D) Cell morphology visualized by microscopy 3 days after induction of *AVR1* or *AVR1*^*ΔT*^ expression with β-estradiol on BCD medium. Arrowheads point to aberrant cell morphology. Scale bars represent 10 μm. (E) Frequency of aberrant cell morphologies observed in the experiment described in D (n >70 cells). Significant differences are indicated by asterisks (P>0.05).

To assess the effects of AVR1 on *P*. *patens*, we monitored a large number of independent AVR1 and AVR1^ΔT^ *P*. *patens* lines on medium with and without β-estradiol (Fig 3). Compared to non-induced lines, *AVR1*-expressing *P*. *patens* lines showed reduced colony sizes and the conspicuous hairy appearance was far less prominent (mild phenotype) or even absent (severe phenotype) (Fig 3B and 3C). Remarkably, moss lines expressing *AVR1*^*ΔT*^ showed the same phenotype. Such aberrant growth phenotypes are caused by a reduction of caulonema cells, which are fast-growing protonema cells with poorly developed chloroplasts [39]. Besides the reduced colony size, we also observed defects in cell morphology (Fig 3D). Chloronema cells of *AVR1* or *AVR1*^*ΔT*^ expressing lines consistently showed abnormal swelling (Fig 3D and 3E). It should be noted that the control recipient moss line, without an *AVR1* or *AVR1*^*ΔT*^ transgene, also showed some reduction in colony size upon transfer to plates containing β-estradiol, but these reductions were very limited when compared to the severe growth reduction observed in the transgenic lines. We suspect that this slight growth reduction is due to some minor damage caused by the transfer itself and not by β-estradiol. Moreover, Kubo et al. [30] who developed the β-estradiol inducible expression vector pPGX8 that we used in this study, did not observe developmental defects in *P*. *patens* lines carrying a GFP-GUS marker gene in pPGX8, and also in other studies using the same system for inducing RNAi silencing or overexpression, the phenotypes differ from the ones we observe in *AVR1* or *AVR1*^*ΔT*^ expressing *P*. *patens* lines [28, 40]. Taken together, these results show that the presence of AVR1 or AVR1^ΔT^ inside the cell disturbs *P*. *patens* cell growth and development.

The finding that the presence of either AVR1 or AVR1^ΔT^ affects moss development in a similar way could be interpreted in two ways. If we assume that AVR1^ΔT^ does not bind PpSec5s *in planta*, as predicted from the Y2H assay, then it is unlikely that the growth phenotype is caused by direct interaction with Sec5. In that case both AVR1 and AVR1^ΔT^ potentially interfere with other processes involved in tip growth. It is not uncommon that a pathogen effector targets multiple host proteins and this could be true for AVR1 as well [25, 41]. In fact, in co-immunoprecipitation experiments we found that AVR1 interacts with several proteins in *N*. *benthamiana*, including multiple clathrin heavy chain homologs (unpublished data). Clathrin-mediated endocytotic membrane trafficking is essential for plant development and tip cell growth by balancing exocytosis [42] and this could explain the cell growth defect in *P*. *patens* caused by AVR1. However, unlike AVR1, AVR1^ΔT^ does not suppress cell death induced by CRINKLERs, another class of *P*. *infestans* effectors, and it does not promote *P*. *infestans* colonization [15] making it less likely that the host targets of AVR1 and AVR1^ΔT^ completely overlap. The second option is that AVR1^ΔT^ does bind to PpSec5, but with a lower affinity than AVR1. Although negative in the Y2H assay, we cannot exclude that *in planta* conditions are more suitable to facilitate interaction between AVR1^ΔT^ and PpSec5. If that is the case, the highly similar phenotypes in the independent *P*. *patens* lines expressing *AVR1* or *AVR1*^*ΔT*^ indicate that capturing Sec5 causes defects in cell growth and development.

### *Sec5*- or *Sec6*-silenced *P*. *patens* lines are phenocopies of *AVR1*-expressing lines

**Fig 4:**
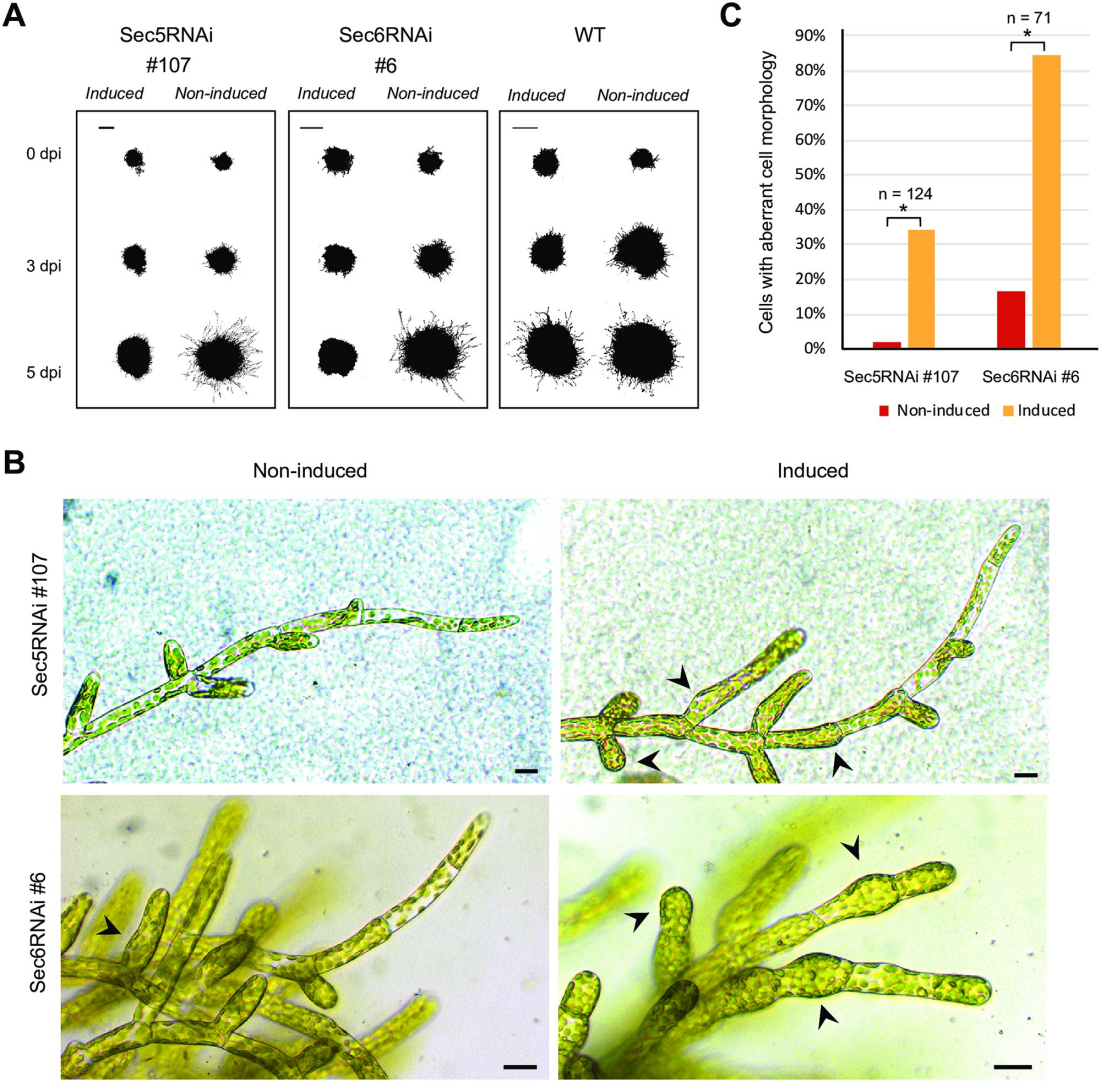
*Sec5-* or *Sec6-*silencing impairs *Physcomitrium patens* growth and development. (A) *P*. *patens* Sec5RNAi and Sec6RNAi lines were grown on BCDAT medium with (induced) and without (non-induced) β-estradiol. The images show colony growth of representative transformants at 0, 3 and 5 days after induction (dpi). This experiment was repeated three times with comparable results. Scale bars represent 2 mm. (B) Cell morphology visualized by microscopy 3 days after induction of *Sec5*- or *Sec6*-silencing with β-estradiol on BCD medium. Arrowheads point to aberrant cell morphology. Scale bars represent 10 μm. (C) Frequency of aberrant cell morphologies observed in the experiment described in B (n >70 cells). Significant differences are indicated by asterisks (P>0.05).

Since AVR1 disrupts Sec5 functioning in potato [15], we anticipated that downregulation of *Sec5* would cause the same effect as expression of AVR1. We therefore made a β-estradiol inducible RNAi construct that targets transcripts of all four *Sec5* homologs of *P*. *patens* for degradation (S3A Fig) and introduced this into the neutral *P*. *patens* PIG1 locus via homologous recombination [31]. The knock-down levels in stable transformed moss lines were validated by quantitative RT-PCR and this showed a reduction of about 40% in gene expression for all four *PpSec5* homologs (S3B Fig). *Sec5*-silencing resulted in reduced caulonemal growth (Fig 4A) and chloronemal tissue with swollen cells (Fig 4B). This swelling may be a consequence of disrupted polarized growth. Disruption of polarized cell growth is a defect that has been associated with exocyst functioning in e.g. pollen tubes and root hairs in seed plants [43, 44], thus pointing to impairment of exocyst functioning in *Sec5*-silenced lines. The phenotypic similarities between *AVR1*-expressing and *Sec5-*silenced *P*. *patens* lines (compare Figs 3 and 4), suggests that the presence of AVR1 disrupts Sec5 functioning.

To test whether the observed defects in *Sec5-*silenced lines are caused by the function of Sec5 as part of the exocyst complex or by exocyst-independent roles of Sec5, we compared the phenotypes of *Sec5-*silenced lines with that of a *Sec6*-silenced line. Sec6 is also part of the exocyst complex and involved in the cytokinesis of *P*. *patens* [28]. Consistent with the phenotype of *Sec5-*silenced lines, we observed that silencing of *Sec6* results in reduced colony growth, absence of caulonemal cells and swelling of chloronema cells (Fig 4) [28]. Others studying *P*. *patens* Exo70.3d-mutants [45] or *Sec10-*silenced *P*. *patens* lines [46], observed similar aberrant phenotypes in polarized tip growth and caulonema development. Taken together this shows that defects in caulonemal development and polarized cell growth are consistent in *P*. *patens* lines in which individual exocyst subunits are lacking or strongly reduced, pointing to malfunctioning of the exocyst. Therefore, we conclude that *Sec5*-silencing leads to (partial) disruption of the exocyst.

### AVR1 does not alter Sec5 localization

**Fig 5:**
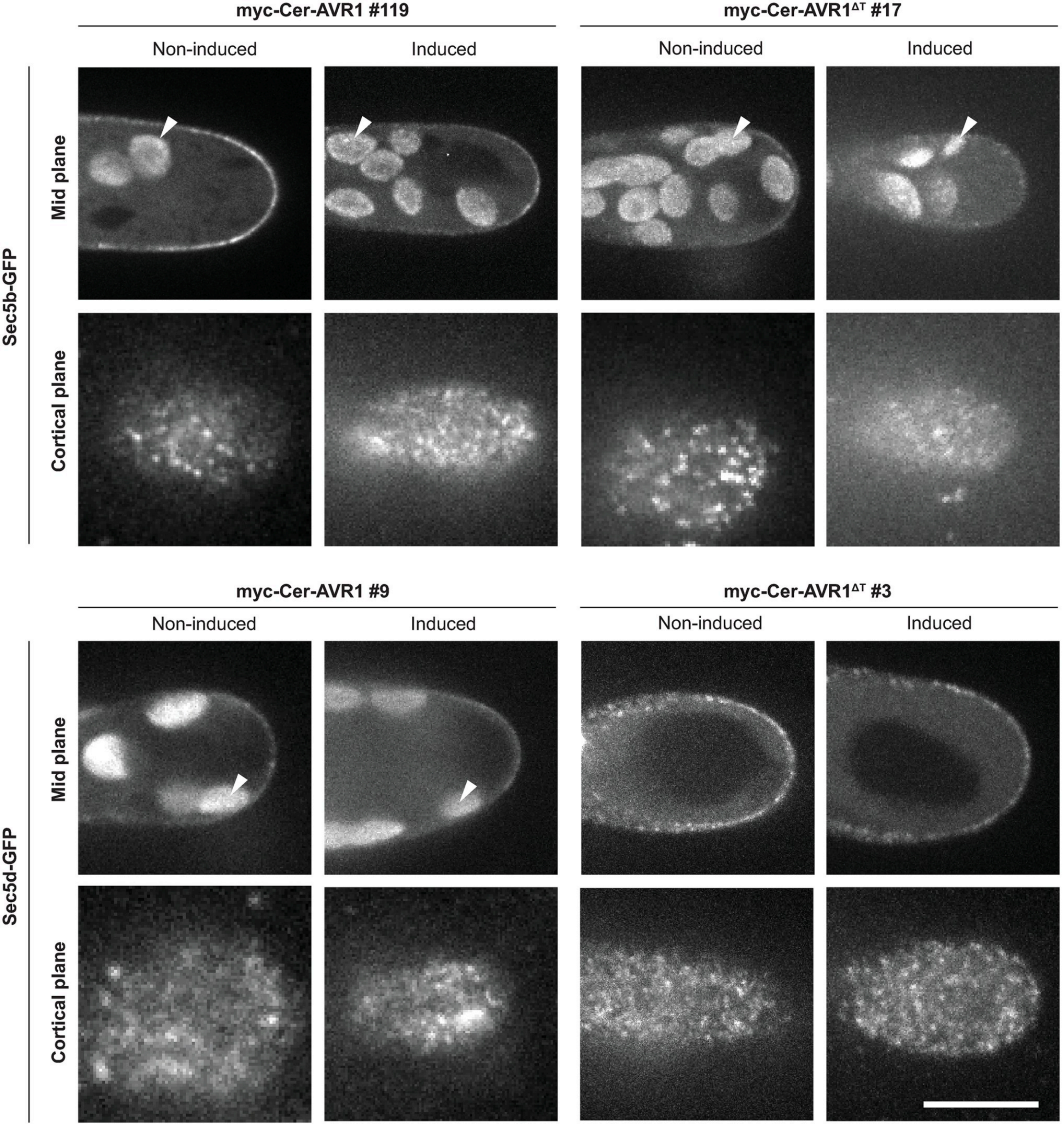
AVR1 and AVR1^ΔT^ do not alter Sec5 the subcellular localization in *Physcomitrium patens*. Subcellular localization of Sec5b-GFP and Sec5d-GFP in absence (no-induced) or presence (induced) of AVR1 or AVR^ΔT^, visualised by confocal fluorescence microscopy. Laser and filter settings were adjusted for excitation of GFP. Mid planes are maximum z-projections of 2.5 μm and cortical planes are average z-projections of 1.5 μm. Auto-fluorescence of chloroplasts (exemplified by arrowheads) is visible inside cells (mid plane). Scale bar represents 10 μm.

To address the question what happens with Sec5 upon AVR1 binding, we investigated the subcellular localization of PpSec5b and PpSec5d in the presence and absence of AVR1. We hypothesized that binding of AVR1 to Sec5 would alter the localization of Sec5 thereby disturbing its role in the exocyst complex. As a first step, we attempted to check whether AVR1 co-localizes with Sec5 in *P*. *patens* caulonema cells. Unfortunately, the expression of *AVR1* upon induction with β-estradiol is very high (Fig 2) and too high to determine whether AVR1 localizes to typical exocyst foci on the PM [43, 46–50]. We then investigated whether AVR1 alters the localization of Sec5b-GFP and Sec5d-GFP. After induction of *AVR1* expression, we observed some variation in intensity of fluorescent foci at the PM, but the localization did not change; both Sec5b-GFP and Sec5d-GFP were visible as characteristic foci at the PM and this pattern was similar in absence of AVR1 (Fig 5).

**Fig 6:**
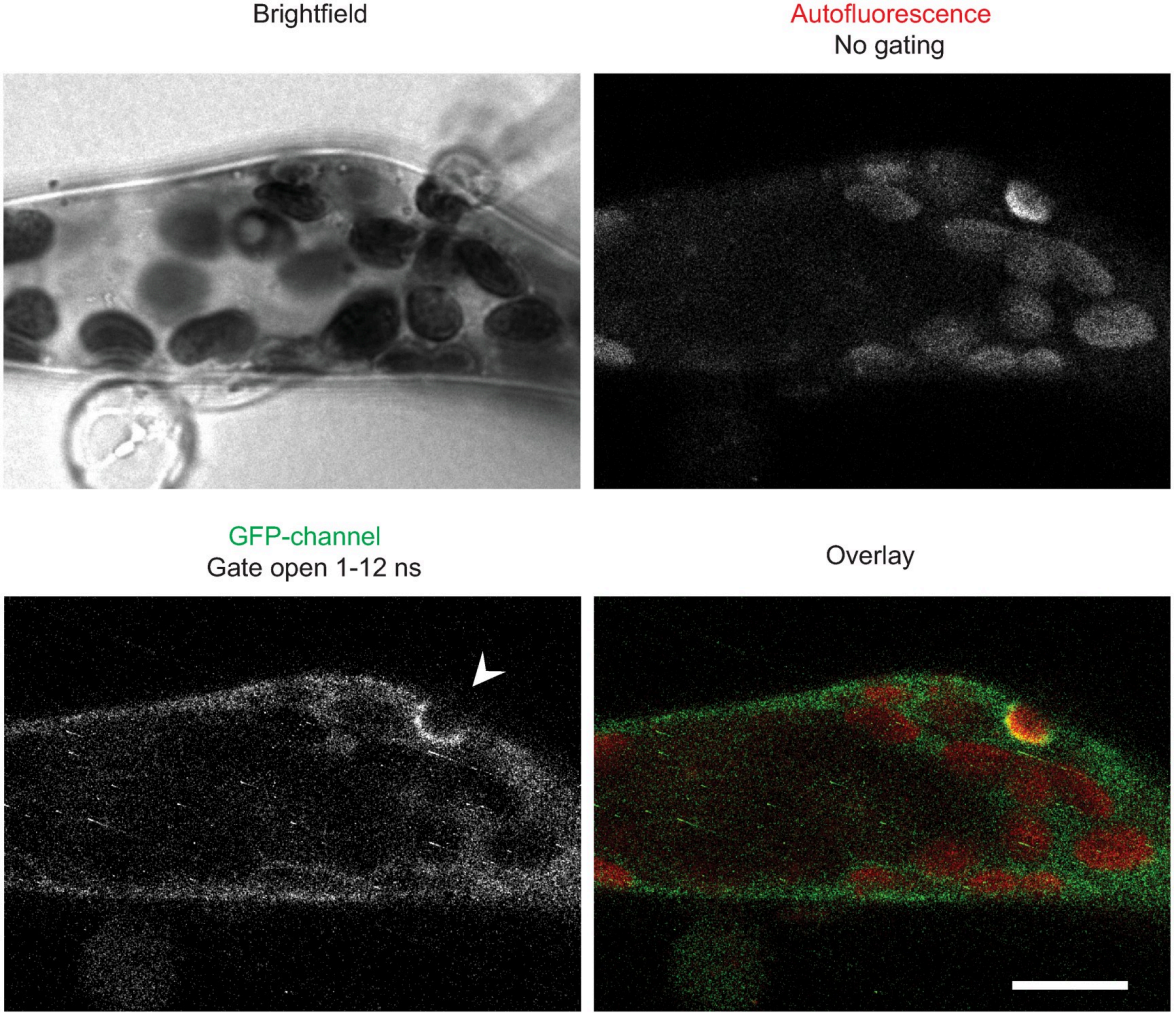
AVR1 does not disrupt Sec5b accumulation or deposition of autofluorescent material at infection sites. Sec5b-GFP accumulation on the membrane surrounding a papilla-like structure (arrowhead) in moss expressing *AVR1* and inoculated with *P*. *capsici*. *AVR1* expression in moss line myc-Cer-AVR1 #119 was induced by growth on BCD medium with β-estradiol for 3 days prior to inoculation. Image shown is a single confocal plane taken 3 hours after inoculation. Scale bar represents 10 μm.

When attacking potato, *P*. *infestans* secretes a large set of RXLR effectors that are deposited inside the plant cell. AVR1 is one of these effectors that normally end up inside the plant cell during pathogen attack. We recently showed that in *P*. *patens* several exocyst subunits, including Sec5b and Sec5d, accumulate at *Phytophthora* infection sites [29]. Because of its binding to Sec5, AVR1 might play a role in redirecting this exocyst subunit. However, the *Phytophthora* species that was used in that study is *Phytophthora capsici*, a species that lacks an AVR1 homolog. It is likely that *P*. *capsici* produces other RXLR effectors that also target exocyst subunits and that could be involved in redirecting the exocyst. To test whether AVR1 can disrupt or enhance recruitment of Sec5 to infection sites, we inoculated a *P*. *patens* line expressing *AVR1* with *P*. *capsici* and studied the localization of Sec5b in infected cells. To improve the visualization, we used time-gated confocal microscopy, a technique that eliminates undesired autofluorescence caused by deposition of phytoalexins or phenolic compounds at infection sites [29]. However, despite this improved visualization we found no indications that AVR1 affects the accumulation of Sec5b-GFP around infection sites. The localisation pattern of Sec5 is similar with or without AVR1 and also deposition of papilla-like structures, a process facilitated by the exocyst as mediator of vesicle trafficking, seems to take place as usual (Fig 6). This suggests that under these circumstances AVR1 is not able to significantly suppress exocytosis despite the fact that AVR1 was shown to be a strong virulence factor [15].

## Conclusion

The cell growth defects of *P*. *patens* lines expressing *AVR1* combined with the fact that this phenotype is copied in *Sec5*- or *Sec6*-silenced lines, is in line with our previous findings in seed plants where AVR1 was shown to disrupt exocyst functioning by binding to Sec5 [15]. However, AVR1 did not alter the localization of Sec5, suggesting that in *P*. *patens* the effect of AVR1 on exocyst functioning is subtle, for instance by slowing down exocyst dynamics or reducing exocyst efficiency. Attempts to quantify Sec5 dynamics by confocal fluorescence microscopy were unsuccessful, and it seems we need more advanced tools to determine how AVR1 modulates Sec5 functioning. *Phytophthora* species secrete a large number of effectors and it is known that pathogens need multiple effectors in order to effectively reduce plant defense [2, 41]. Therefore, it is logical to assume that, besides AVR1, more effectors will be identified that target exocyst subunits and/or affect their subcellular localization. In potato and *N*. *benthamiana*, AVR1 acts as a virulence factor by promoting *P*. *infestans* colonization and suppressing host defense responses such as the deposition of callose [15]. To answer the question whether AVR1 is also able to suppress exocytosis to penetration attempt sites requires a precise quantification of the amount of deposited autofluorescent material at these sites in the presence and absence of AVR1.

## Supporting information

S1 Fig Autoactivation tests of Y2H bait and prey proteins. (TIF)

S2 Fig Generation and genotyping of *Physcomitrium patens* lines expressing *AVR1-* and *AVR1*^*ΔT*^. (A) Schematic representation of the *AVR1* and *AVR1*^*ΔT*^ pPGX8 constructs that were transformed into *P*. *patens* for homologous recombination at the PIG1 neutral locus. PIG1bR/L: DNA fragments flanking the PIG1 neutral locus. LexA:Pm35S: bacterial LexA operator fused to a CaMV minimal 35S promoter. Term: terminator. GX8: promoter of a constitutively expressed *P*. *patens* gene. XVE: a sequence encoding a chimeric transcription activator composed of the DNA-binding domain of the bacterial repressor LexA, the transcriptional activation domain VP16 and the C-terminal region of the human estrogen receptor. hygR: hygromycin resistance cassette driven by a modified CaMV35S promoter. Upon β-estradiol treatment, the XVE protein binds to the LexA operator and induces the expression of the downstream gene resulting in the production of myc and/or cerulean tagged AVR1 or AVR1^ΔT^ [30]. The location of the primers used for genotyping is indicated in red and primer sequences are listed in S2 Table in S1 File. 1: primer EO117, 2: EO121, 3: EO122, 4: EO120, 5: JK130 and 6: EO114. (B) PCR analyses of *P*. *patens* transformants to confirm homologous recombination at the PIG1 neutral locus. Each construct shown in (A) was transformed into two recipient *P*. *patens* lines (background), one containing Sec5b-GFP and the other containing Sec5d-GFP. PCR fragments are visualized by agarose gel electrophoresis. Line numbers of independent transformants (#) and primers sets with predicted sizes of the PCR fragments in brackets (bp: base pairs) are indicated.
(TIF)

S3 Fig Generation of Sec5RNAi *Physcomitrium patens* lines. (A) Schematic representation of the *Sec5* silencing construct. PIG1bR/L: DNA fragments for homologous recombination into the PIG1 neutral locus. LexA:Pm35S: bacterial LexA operator fused to a CaMV minimal 35S promoter. Term: terminator. pKINID1a: promoter of a constitutively expressed *P*. *patens* gene. XVE: a sequence encoding a chimeric transcription activator composed of the DNA-binding domain of the bacterial repressor LexA, the transcriptional activation domain VP16 and the C-terminal region of the human estrogen receptor. hygR: hygromycin resistance cassette driven by a modified CaMV35S promoter and terminator. Upon β-estradiol treatment, the XVE protein binds to the LexA operator and induces the expression of the *Sec5*-silencing fragment. Backbone of the construct is vector pGG626 [31]. (B) Expression analyses by quantitative RT-PCR. Relative expression of *P*. *patens Sec5a*, *Sec5b*, *Sec5c* and *Sec5d* in independent Sec5RNAi moss lines two days after growth on medium with (+) and without (-) β-estradiol. Transcript levels were normalized to *PpEF1α* transcript levels and expressed as mean fold changes (± standard deviation) relative to the transcript level in non-induced moss tissue (set at 1). Statistical differences are indicated by asterisks (P>0.05). Background indicates the recipient strain used for transformation of the Sec5 silencing construct.
(TIF)

S4 Fig Alignment of the four *Physcomitrium patens* Sec5 proteins and *Solanum tuberosum* Sec5-1. Forty-one amino acids that are shared between StSec5, PpSec5b and PpSec5d but different in PpSec5a and PpSec5c are indicated with arrowheads. Colors shadings distinguish amino acids that are hydrophobic (skyblue), positively charged (red), negatively charged (magenta), polar (applegreen) or aromatic (cyan). Cysteines are colored pink, glycines bronze and prolines lime. Amino acids that are not conserved are not color shaded.
(TIF)

S1 File (PDF)

S1 Raw images (PDF)

## References

[1] Kroon LP, Brouwer H, de Cock AW, Govers F. The genus *Phytophthora* anno 2012. Phytopathology 2012; 102:348–364. 10.1094/PHYTO-01-11-0025 22185336

[2] Haas BJ, Kamoun S, Zody MC, Jiang RH, Handsaker RE, Cano LM, et al. Genome sequence and analysis of the Irish potato famine pathogen *Phytophthora infestans*. Nature 2009; 461:393–398. 10.1038/nature08358 19741609

[3] Stassen JHM, van den Ackerveken G. How do oomycete effectors interfere with plant life? Curr Opin Plant Biol. 2011; 14,407–414. 10.1016/j.pbi.2011.05.002 21641854

[4] Bouwmeester K, van Poppel PMJA, Govers F. Genome biology cracks enigmas of oomycete plant pathogens. In: Parker J., editor. Annual Plant Reviews 34, Molecular Aspects of Plant Disease Resistance. Wiley-Blackwell; 2009. pp. 102–134.

[5] Whisson SC, Boevink PC, Wang S, Birch PR. The cell biology of late blight disease. Curr Opin in Microbiol. 2016; 34:127–135. 10.1016/j.mib.2016.09.002 27723513 PMC5340842

[6] Bos JIB, Kanneganti TD, Young C, Cakir C, Huitema E, Win J, et al. The C-terminal half of *Phytophthora infestans* RXLR effector AVR3a is sufficient to trigger R3a-mediated hypersensitivity and suppress INF1-induced cell death in *Nicotiana benthaminana*. Plant J. 2006; 48: 165–176. 10.1111/j.1365-313X.2006.02866.x 16965554

[7] van Poppel PMJA, Guo J, van der Vondervoort PJIV, Jung MWM, Birch PRJ, Whisson SC, et al. The *Phytophthora infestans* avirulence gene Avr4 encodes an RXLR-dEER effector. Mol Plant Microbe In. 2008; 21:1460–1470. 10.1094/MPMI-21-11-1460 18842095

[8] Jiang RHY, Tripathy S, Govers F, Tyler BM. RXLR effector reservoir in two *Phytophthora* species is dominated by a single rapidly evolving superfamily with more than 700 members. Proc Natl Acad Sci USA 2008; 105:4874–4879. 10.1073/pnas.0709303105 18344324 PMC2290801

[9] Champouret N, Bouwmeester K, Rietman H, van der Lee T, Maliepaard C, Heupink A, et al. *Phytophthora infestans* isolates lacking class I ipiO variants are virulent on Rpi-blb1 potato. Mol Plant Microbe In. 2009; 22:1535–1545. 10.1094/MPMI-22-12-1535 19888819

[10] Bouwmeester K, de Sain M, Weide R, Gouget A, Klamer S, Canut H, et al. The lectin receptor kinase LecRK-I.9 is a novel *Phyophthora* resistance component and a potential host target for a RXLR effector. PLoS Pathog. 2011; 7:e1001327. 10.1371/journal.ppat.1001327 21483488 PMC3068997

[11] Bos JIB, Armstrong MR, Gilroy EM, Boevink PC, Hein I, Taylor RM, et al. *Phytophthora infestans* effector AVR3a is essential for virulence and manipulates plant immunity by stabilizing host E3 ligase CMPG1. Proc Natl Acad Sci USA 2010; 107:9909–9914. 10.1073/pnas.0914408107 20457921 PMC2906857

[12] McLellan H, Boevink PC, Armstrong MR, Pritchard L, Gomez S, Morales J, et al. An RxLR effector from *Phytophthora infestans* prevents re-localisation of two plant NAC transcription factors from the endoplasmic reticulum to the nucleus. PLoS Pathog. 2013; 9:e1003670. 10.1371/journal.ppat.1003670 24130484 PMC3795001

[13] King SR, McLellan H, Boevink PC, Armstrong MR, Bukharova T, Sukarta O, et al. *Phytophthora infestans* RXLR effector PexRD2 interacts with host MAPKKKε to suppress plant immune signaling. Plant Cell 2014; 26:1345–1359. 10.1105/tpc.113.120055 24632534 PMC4001388

[14] Jones JD, Dangl JL. The plant immune system. Nature 2006; 444:323–329. 10.1038/nature05286 17108957

[15] Du Y, Mpina MH, Birch PRJ, Bouwmeester K, Govers F. *Phytophthora infestans* RXLR effector AVR1 interacts with exocyst component Sec5 to manipulate plant immunity. Plant Physiol. 2015; 169:1975–1990. 10.1104/pp.15.01169 26336092 PMC4634092

[16] Du Y, Berg J, Govers F, Bouwmeester K. Immune activation mediated by the late blight resistance protein R1 requires nuclear localization of R1 and the effector AVR1. New Phytol. 2015; 207:735–747. 10.1111/nph.13355 25760731

[17] Du Y, Weide R, Zhao Z, Msimuko P, Govers F, Bouwmeester K. RXLR effector diversity in *Phytophthora infestans* isolates determines recognition by potato resistance proteins; the case study AVR1 and R1. Stud Mycol. 2018; 89:85–93. 10.1016/j.simyco.2018.01.003 29910515 PMC6002335

[18] Ravikumar R, Steiner A, Assaad FF. Multisubunit tethering complexes in higher plants. Curr Opin Plant Biol. 2017; 40:97–105. 10.1016/j.pbi.2017.08.009 28889036

[19] Žárský V, Kulich I, Fendrych M, Pečenková T. Exocyst complexes multiple functions in plant cells secretory pathways. Curr Opin Plant Biol. 2013; 16, 726–733. 10.1016/j.pbi.2013.10.013 24246229

[20] Martin-Urdiroz M, Deeks MJ, Horton CG, Dawe HR, Jourdain I. The exocyst complex in health and disease. Front Cell Dev Biol. 2016; 4:24, 10.3389/fcell.2016.00024 27148529 PMC4828438

[21] Du Y, Overdijk EJR, Berg JA, Govers F, Bouwmeester K. Solanaceous exocyst subunits are involved in immunity to diverse plant pathogens. J Exp Bot. 2018; 69:655–666. 10.1093/jxb/erx442 29329405 PMC5853398

[22] Kulich I, Vojtíková Z, Sabol P, Ortmannová J, Neděla V, Tihlaříková E, et al. Exocyst subunit Exo70H4 has a specific role in callose synthase secretion and silica accumulation. Plant Physiol. 2018; 176:2040–2051. 10.1104/pp.17.01693 29301954 PMC5841730

[23] Guo J, Xu C, Wu D, Zhao Y, Qiu Y, Wang X, et al. Bph6 encodes an exocyst-localized protein and confers broad resistance to planthoppers in rice. Nat Genet. 2018; 10.1038/s41588-018-0039-6 29358653

[24] Fujisaki K, Abe Y, Ito A, Saitoh H, Yoshida K, Kanzaki H, et al. Rice Exo70 interacts with a fungal effector, AVR-Pii, and is required for AVR-Pii-triggered immunity. Plant J. 2015; 83:875–887. 10.1111/tpj.12934 26186703

[25] Wessling R, Epple P, Altmann S, He Y, Yang L, Henz SR, et al. Convergent targeting of a common host protein-network by pathogen effectors from three kingdoms of life. Cell Host Microbe 2014; 16:365–375. 10.1016/j.chom.2014.08.004 25211078 PMC4191710

[26] Overdijk EJR, de Keijzer J, de Groot D, Schoina C, Bouwmeester K, Ketelaar T, et al. Interaction between the moss *Physcomitrella* patens and *Phytophthora*: a novel pathosystem for live-cell imaging of subcellular defence. J Microsc 2016; 263:171–180. 10.1111/jmi.12395 27027911

[27] Carella P, Schornack S. Manipulation of bryophyte hosts by pathogenic and symbiotic microbes. Plant Cell Physiol 2018; 59:656–665. 10.1093/pcp/pcx182 29177478 PMC6018959

[28] Tang H, de Keijzer J, Overdijk EJR, Sweep E, Steentjes M, Vermeer J, et al. Exocyst subunit Sec6 is positioned by microtubule overlaps in the moss phragmoplast prior to the arrival of cell plate membrane. J Cell Sci. 2019; 10.1242/jcs.222430 30635445

[29] Overdijk EJR, Tang H, Borst JW, Govers F, Ketelaar T. Time‐gated confocal microscopy reveals accumulation of exocyst subunits at the plant‐pathogen interface. J Exp Bot. 2020; 71:837–849. 10.1093/jxb/erz478 31665494

[30] Kubo M, Imai A, Nishiyama T, Ishikawa M, Sato Y, Kurata T, et al. System for stable beta-estradiol-inducible gene expression in the moss *Physcomitrella patens*. PLoS One 2013; 8: e77356. 10.1371/journal.pone.0077356 24086772 PMC3785464

[31] Nakaoka Y, Miki T, Fujioka R, Uehara R, Tomioka A, Obuse C, et al. An inducible RNA interference system in *Physcomitrella patens* reveals a dominant role of augmin in phragmoplast microtubule generation. Plant Cell 2012; 24:1478–1493. 10.1105/tpc.112.098509 22505727 PMC3398558

[32] James P, Halladay J, Craig EA. Genomic libraries and a host strain designed for highly efficient two-hybrid selection in yeast. Genetics 1996; 144:1425–1436. 8978031 10.1093/genetics/144.4.1425 PMC1207695

[33] Nishiyama T, Hiwatashi Y, Sakakibara I, Kato M, Hasebe M. Tagged mutagenesis and gene-trap in the moss, *Physcomitrella patens* by shuttle mutagenesis. DNA Res. 2000; 7:9–17. 10.1093/dnares/7.1.9 10718194

[34] Ashton NW, Cove DJ. Isolation and preliminary characterization of auxotrophic and analog resistant mutants of moss, *Physcomitrella patens*. Molecular and General Genetics 1977; 154; 87–95.

[35] Yamada M, Miki T, Goshima G. Imaging mitosis in the moss *Physcomitrella patens*. In: Chang P, Ohi R, editors. Methods in Molecular Biology. Springer New York; 2016. pp. 263–282. 10.1007/978-1-4939-3542-0_17 27193855

[36] Livak KJ, Schmittgen TD. Analysis of relative gene expression data using real-time quantitative PCR and the 2(-Delta Delta C(T)). Methods 2001; 25:402–408. 10.1006/meth.2001.1262 11846609

[37] Schindelin J, Arganda-Carreras I, Frise E, Kaynig V, Longair M, Pietzsch T, et al. Fiji: an open-source platform for biological-image analysis. Nat Methods 2012; 9:676–682. 10.1038/nmeth.2019 22743772 PMC3855844

[38] Wang S, McLellan H, Bukharova T, He Q, Murphy F, Shi J, et al. *Phytophthora infestans* RXLR effectors act in concert at diverse subcellular localisations to enhance host colonisation. J Exp Bot. 2018; 70:343–356. 10.1093/jxb/ery360 PMC6305197 30329083

[39] Menand B, Calder G, Dolan L. Both chloronemal and caulonemal cells expand by tip growth in the moss *Physcomitrella patens*. J Exp Bot. 2007; 58:1843–1849. 10.1093/jxb/erm047 17404383

[40] Kosetsu K, de Keijzer J, Janson ME, Goshima G. MICROTUBULE-ASSOCIATED PROTEIN65 is essential for maintenance of phragmoplast bipolarity and formation of the cell plate in *Physcomitrella patens*. The Plant Cell 2013; 25:4479–4492. 10.1105/tpc.113.117432 24272487 PMC3875731

[41] Khan M, Seto D, Subramaniam R, Desveaux D. Oh, the places they’ll go! A survey of phytopathogen effectors and their host targets. Plant J. 2018; 93:651–663. 10.1111/tpj.13780 29160935

[42] Grebnev G, Ntefidou M, Kost B. Secretion and endocytosis in pollen tubes: models of tip growth in the spot light. Front Plant Sci. 2017; 8: 10.3389/fpls.2017.00154 28224002 PMC5293803

[43] Bloch D, Pleskot R, Pejchar P, Potochký M, Trpkošová P, Cwiklik L, et al. Exocyst Sec3 and phosphoinositides define sites of exocytosis and pollen tube initiation and growth. Plant Physiol. 2016; 172:980–1002. 10.1104/pp.16.00690 27516531 PMC5047084

[44] Sekereš J, Pejchar P, Šantrůček J, Vukašinović N, Žárský V, Potocký M. Analysis of exocyst subunit Exo70 family reveals distinct membrane domains in tobacco pollen tubes. Plant Physiol. 2017; pp.01709.2016. 10.1104/pp.16.01709 28082718 PMC5338673

[45] Rawat A, Brejšková L, Hála M, Cvrčková F, Žárský V. The *Physcomitrella patens* exocyst subunit Exo70.3d has distinct roles in growth and development, and is essential for completion of the moss life cycle. New Phytol. 2017; 10.1111/nph.14548 28397275

[46] van Gisbergen PAC, Wu S, Chang M, Pattavina KA, Bartlett ME, Bezanilla M. An ancient Sec10–formin fusion provides insights into actin-mediated regulation of exocytosis. J Cell Biol. 2018; 217:945–957. 10.1083/jcb.201705084 29374070 PMC5839782

[47] Zhang Y, Immink R, Liu CM, Emons AM, Ketelaar T. The *Arabidopsis* exocyst subunit Sec3A is essential for embryo development and accumulates in transient puncta at the plasma membrane. New Phytol. 2013; 199:74–88. 10.1111/nph.12236 23495664

[48] Vukašinović N, Oda Y, Pejchar P, Synek L, Pečenková T, Rawat A, et al. Microtubule-dependent targeting of the exocyst complex is necessary for xylem development in *Arabidopsis*. New Phytol. 2016; 213:1052–1067. 10.1111/nph.14267 27801942

[49] Synek L, Vukašinović N, Kulich I, Hála M, Aldorfová K, Fendrych M, et al. Exo70C2 is a key regulatory factor for optimal tip growth of pollen. Plant Physiol. 2017; 174:223–240. 10.1104/pp.16.01282 28356503 PMC5411130

[50] Fendrych M, Synek L, Pecěnková T, Drdová ED, Sekereš J, de Rycke R, et al. Visualization of the exocyst complex dynamics at the plasma membrane of *Arabidopsis* thaliana. Mol Biol Cell 2013; 24:510–520. 10.1091/mbc.E12-06-0492 23283982 PMC3571873

