# Supplementary figures and images for: Phytophthora infestans RXLR effector AVR1 disturbs the growth of Physcomitrium patens without affecting Sec5 localization
Source: PLoS One. 2021 Apr 8;16(4):e0249637. doi: 10.1371/journal.pone.0249637 (PMC8031463; doi:10.1371/journal.pone.0249637)

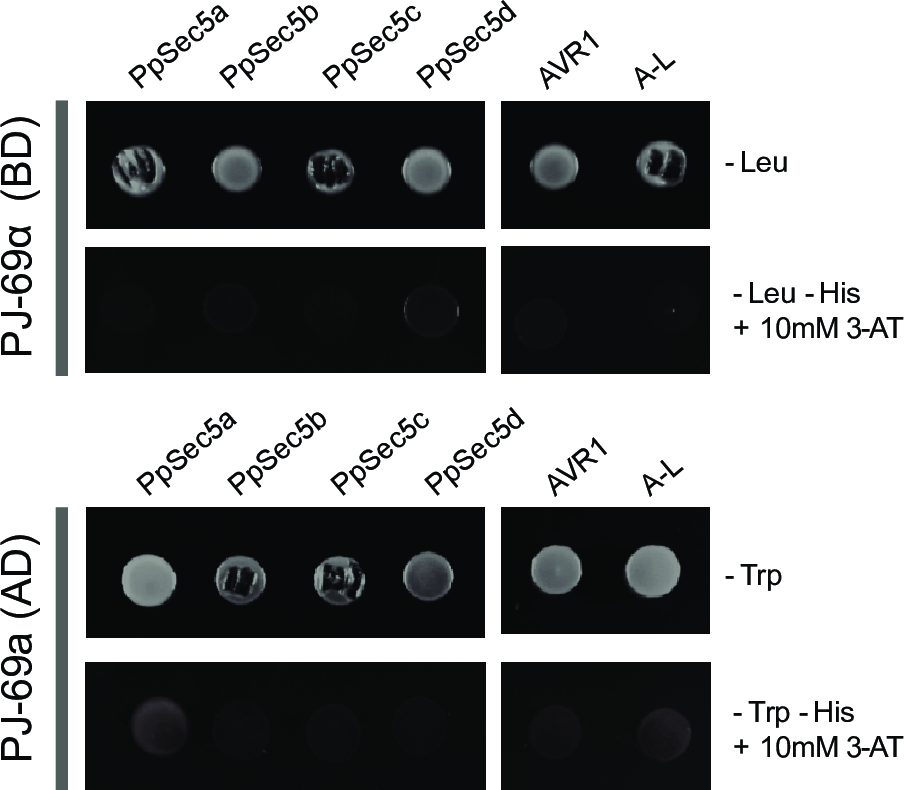

Supplement: S1 Fig — (TIF) [file pone.0249637.s001.tif]

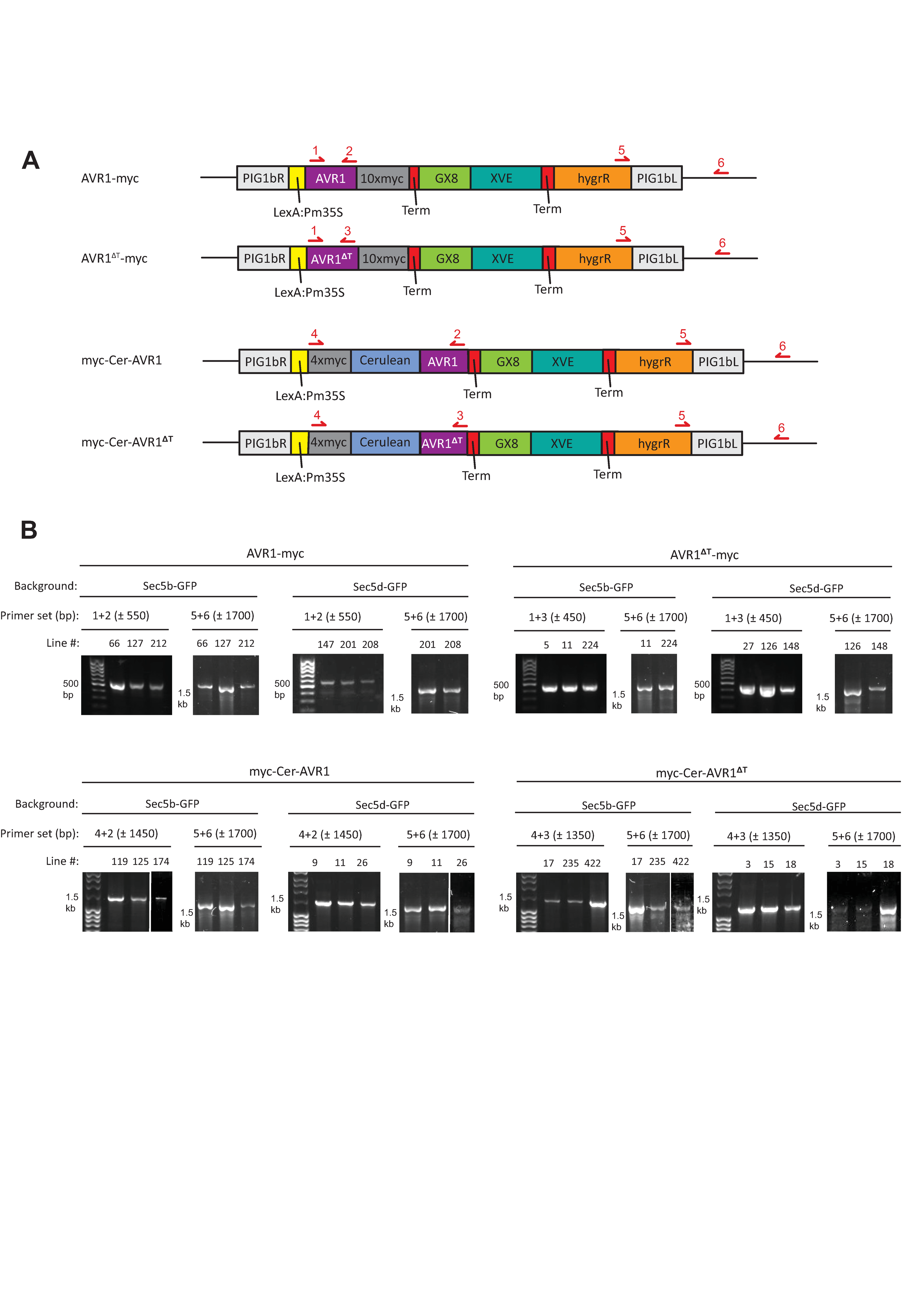

Supplement: S2 Fig — (A) Schematic representation of the AVR1 and AVR1ΔT pPGX8 constructs that were transformed into P. patens for homologous recombination at the PIG1 neutral locus. PIG1bR/L: DNA fragments flanking the PIG1 neutral locus. LexA:Pm35S: bacterial LexA operator fused to a CaMV minimal 35S promoter. Term: terminator. GX8: promoter of a constitutively expressed P. patens gene. XVE: a sequence encoding a chimeric transcription activator composed of the DNA-binding domain of the bacterial repressor LexA, the transcriptional activation domain VP16 and the C-terminal region of the human estrogen receptor. hygR: hygromycin resistance cassette driven by a modified CaMV35S promoter. Upon β-estradiol treatment, the XVE protein binds to the LexA operator and induces the expression of the downstream gene resulting in the production of myc and/or cerulean tagged AVR1 or AVR1ΔT [30]. The location of the primers used for genotyping is indicated in red and primer sequences are listed in S2 Table in S1 File. 1: primer EO117, 2: EO121, 3: EO122, 4: EO120, 5: JK130 and 6: EO114. (B) PCR analyses of P. patens transformants to confirm homologous recombination at the PIG1 neutral locus. Each construct shown in (A) was transformed into two recipient P. patens lines (background), one containing Sec5b-GFP and the other containing Sec5d-GFP. PCR fragments are visualized by agarose gel electrophoresis. Line numbers of independent transformants (#) and primers sets with predicted sizes of the PCR fragments in brackets (bp: base pairs) are indicated. (TIF) [file pone.0249637.s002.tif]

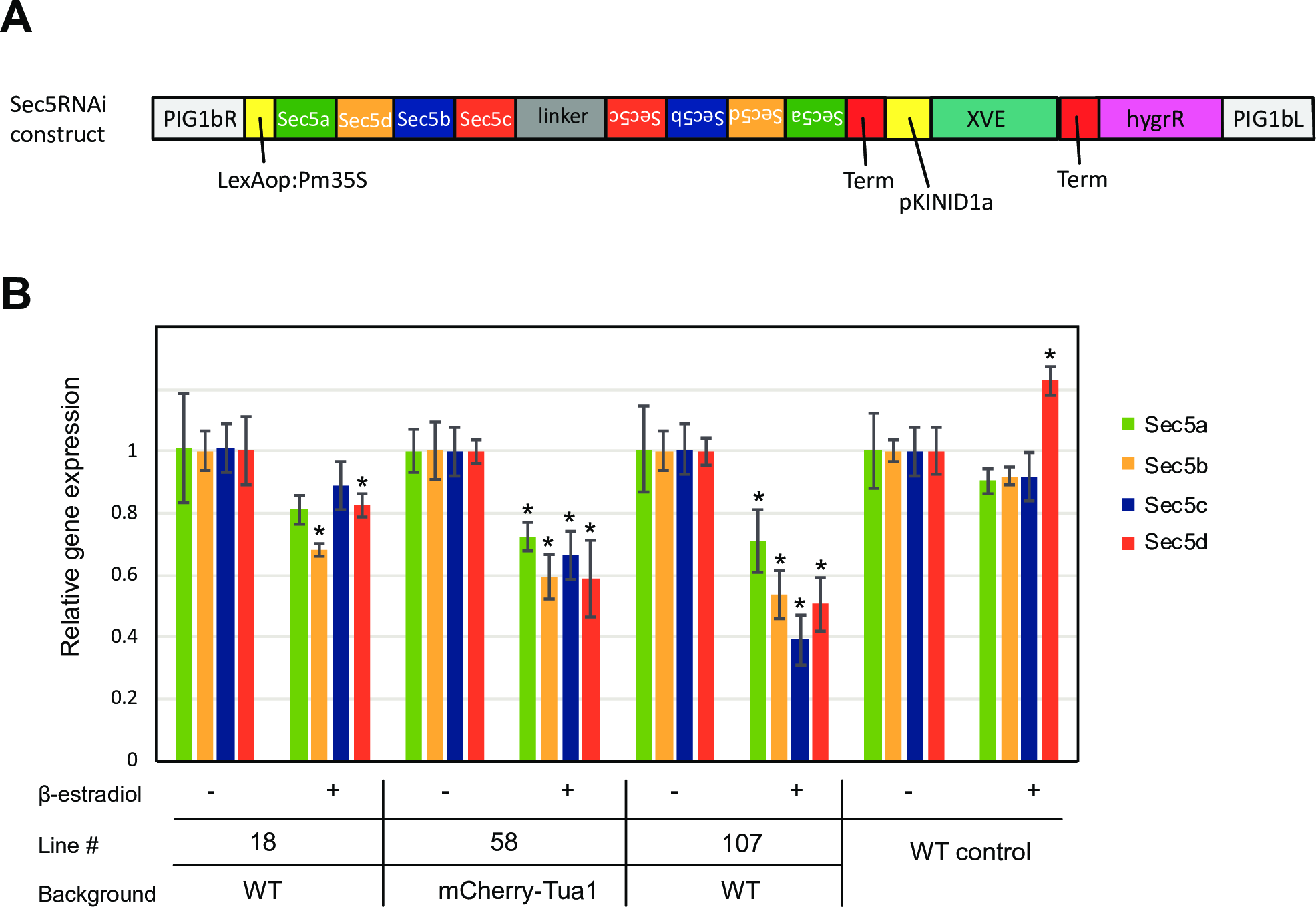

Supplement: S3 Fig — (A) Schematic representation of the Sec5 silencing construct. PIG1bR/L: DNA fragments for homologous recombination into the PIG1 neutral locus. LexA:Pm35S: bacterial LexA operator fused to a CaMV minimal 35S promoter. Term: terminator. pKINID1a: promoter of a constitutively expressed P. patens gene. XVE: a sequence encoding a chimeric transcription activator composed of the DNA-binding domain of the bacterial repressor LexA, the transcriptional activation domain VP16 and the C-terminal region of the human estrogen receptor. hygR: hygromycin resistance cassette driven by a modified CaMV35S promoter and terminator. Upon β-estradiol treatment, the XVE protein binds to the LexA operator and induces the expression of the Sec5-silencing fragment. Backbone of the construct is vector pGG626 [31]. (B) Expression analyses by quantitative RT-PCR. Relative expression of P. patens Sec5a, Sec5b, Sec5c and Sec5d in independent Sec5RNAi moss lines two days after growth on medium with (+) and without (-) β-estradiol. Transcript levels were normalized to PpEF1α transcript levels and expressed as mean fold changes (± standard deviation) relative to the transcript level in non-induced moss tissue (set at 1). Statistical differences are indicated by asterisks (P>0.05). Background indicates the recipient strain used for transformation of the Sec5 silencing construct. (TIF) [file pone.0249637.s003.tif]

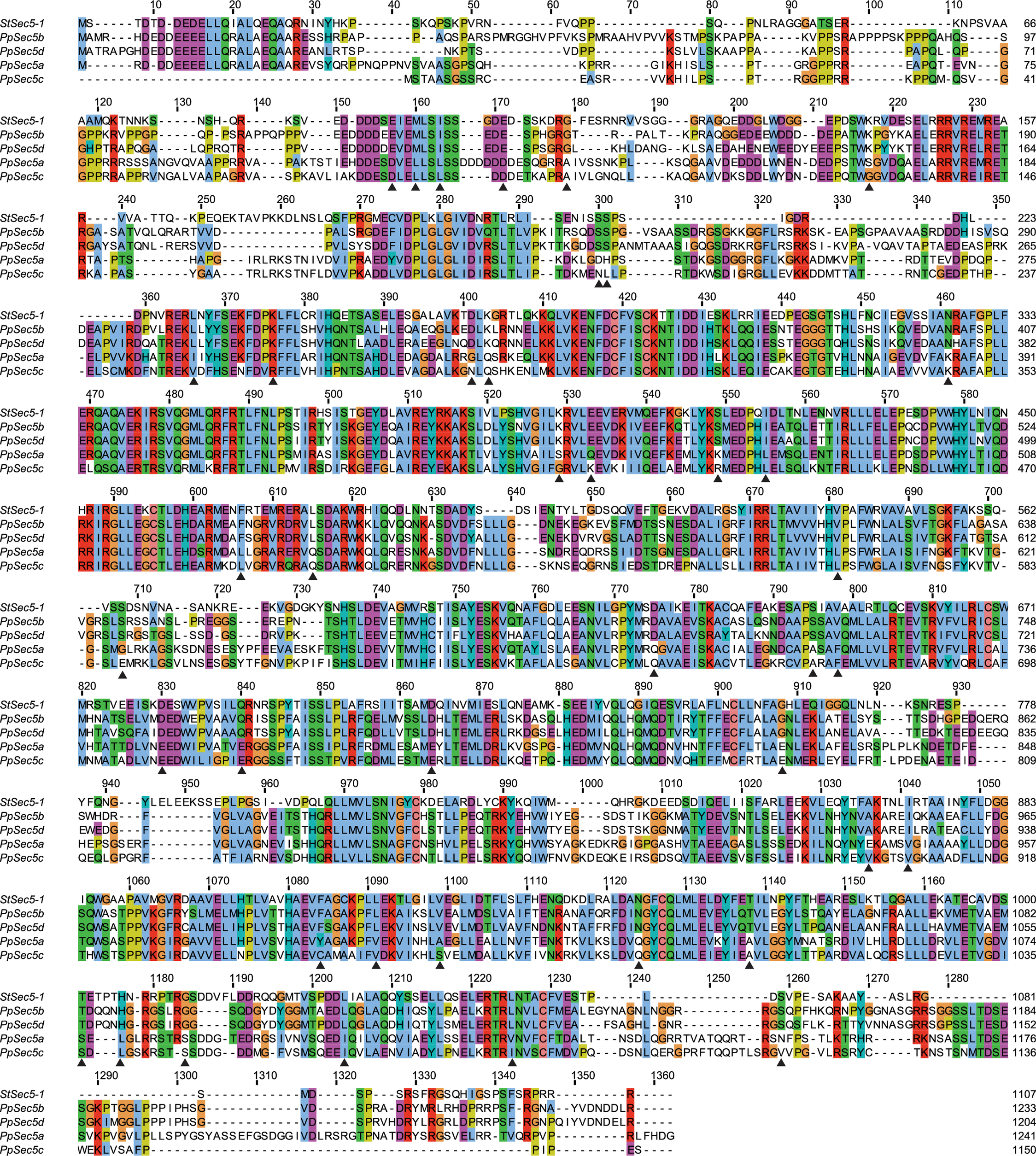

Supplement: S4 Fig — Forty-one amino acids that are shared between StSec5, PpSec5b and PpSec5d but different in PpSec5a and PpSec5c are indicated with arrowheads. Colors shadings distinguish amino acids that are hydrophobic (skyblue), positively charged (red), negatively charged (magenta), polar (applegreen) or aromatic (cyan). Cysteines are colored pink, glycines bronze and prolines lime. Amino acids that are not conserved are not color shaded. (TIF) [file pone.0249637.s004.tif]
