# Supplementary material for: Phytophthora infestans RXLR effector AVR1 disturbs the growth of Physcomitrium patens without affecting Sec5 localization
Source: PLoS One. 2021 Apr 8;16(4):e0249637. doi: 10.1371/journal.pone.0249637 (PMC8031463; doi:10.1371/journal.pone.0249637)
Supplement: S1 File — (PDF) [file pone.0249637.s005.pdf]

Supporting Tables S1-S4

belonging to PlosOne publication entitled

Phytophthora infestans RXLR effector AVR1 disturbs the growth of *Physcomitrium patens* without affecting Sec5 localization

by

Elysa J.R. Overdijk, Vera Putker, Joep Smits, Han Tang, Klaas Bouwmeester, Francine Govers and Tijs Ketelaar

Table S1. Sec5 gene IDs.

Gene	Code	Resource
PpSec5a	Pp1s41_289; Pp3c5_4060	Cossmoss.org
PpSec5b	Pp1s38_295; Pp3c1_7430	Cossmoss.org
PpSec5c	Pp1s18_342; Pp3c24_1100	Cossmoss.org
PpSec5d	Pp1s11_129; Pp3c11_13110	Cossmoss.org
AtSec5a	AT1G76850	Arabidopsis.org
AtSec5b	AT1G21170	Arabidopsis.org
StSec5-1	Sotub04g023510	Solgenomics.net
StSec5-2	Sotub12g024810	Solgenomics.net
ScSec5	P89102	Uniprot.org

Table S2. Primers used in this study.

Primer	Use	Sequence (5' > 3')	Nr
Sec5a-F	Cloning of Sec5a coding sequence for Y2H	CACCATGAGGGACGACGACGAG	2857
Sec5a-R	Cloning of Sec5a coding sequence for Y2H	CTAACCATCATGGAAGAGTCTTGGAAC	EO57
Sec5b-F	Cloning of Sec5b coding sequence for Y2H	CACCATGGCGATGCGACATGACG	2855
Sec5b-R	Cloning of Sec5b coding sequence for Y2H	TTATCTCAAGTCATCGTTATCTACATAGGC	EO58
Sec5c-F	Cloning of Sec5c coding sequence for Y2H	CACCATGTCGACAGCGGCCTCAG	2852
Sec5c-R	Cloning of Sec5c coding sequence for Y2H	CTATGACTCCGGGATGGGAGG	EO59
Sec5d-F	Cloning of Sec5d coding sequence for Y2H	CACCATGGCGACGCGGGCGCC	2860
Sec5d-R	Cloning of Sec5d coding sequence for Y2H	CTATCTCAACTCATCGTTATCTACATAAATTTGAGG	EO60
AVR1-F	AVR1 cloning	ATGTTCGACCACGACAAGG	2072
Start-myc-F	Myc-Cer-AVR1/ΔT cloning	CACCGCAGAATGGGGTTAATTAACGGTGAAC	EO80
Myc-cer-R	Myc-Cer-AVR1/ΔT cloning	CAGCTCCTCGCCCTTGCTCACGCTACCGTTCAAGTCTTCCTC	EO81
Cer-myc-F	Myc-Cer-AVR1/ΔT cloning	GAGGAAGACTTGAACGGTAGCGTGAGCAAGGGCGAGGAGCTG	EO82
Cer-AVR1-R	Myc-Cer-AVR1/ΔT cloning	GGAACCTTGTCGTGGTCGAACTTGTACAGCTCGTCCATGCC	EO83
AVR1-cer-F	Myc-Cer-AVR1/ΔT cloning	GGCATGGACGAGCTGTACAAGTTCGACCACGACAAGGTTCC	EO84
AVR1ΔT-R-wistop	Myc-Cer-AVR1 cloning	TTACCTCCACTTCACAGCCCGTGCC	EO85
AVR1-R-wistop	Myc-Cer-AVR1ΔT cloning	TTAAAATGGTACCACAACATGTCC	EO86
PIG1-locus-R	Genotyping AVR1 moss lines	CCCTCTGCCGACCCATATTTC	EO114
AVR1-koz-F	Genotyping AVR1 moss lines and cloning of AVR1/ΔT-myc	CACCGCAGAATGTTCGACCACG	EO117
10myc-R	Cloning of AVR1/ΔT-myc	GAAATTCGAGCCTAAGCACCGTTC	EO119
AVR1-F-gt	Genotyping AVR1 moss lines	ATGGGGTTAATTAACGGTGAACAAAAGC	EO120
AVR1-R-gt	Genotyping AVR1 moss lines	AAATGGTACCACAACATGTCCACCAAG	EO121
AVR1ΔT-R-gt	Genotyping AVR1 moss lines	CCTCCACTTCACAGCCCGTG	EO122
T35S-F	Genotyping AVR1 moss lines	TGCTAAGGCAGGGTTGGTTACG	JK130
PpSEC5a_RNAi_F_XhoI	Cloning Sec5RNAi construct	CACCCTCGAGCCACCAAATCAGCCGCCT	EO49
PpSEC5a_RNAi_R_SalI	Cloning Sec5RNAi construct	AAAGTCGACCTCTGCTTGATCAACTCCACTCC	EO50
PpSEC5b_RNAi_F_XhoI	Cloning Sec5RNAi construct	CACCCTCGAGAGCAGGCAGCGAGGGAGA	EO51
PpSEC5b_RNAi_R_SalI	Cloning Sec5RNAi construct	AAAGTCGACTTCCAGGTCGCAGGCTCG	EO52
PpSEC5c_RNAi_F_XhoI	Cloning Sec5RNAi construct	CACCCTCGAGCCTGCAAAAACACCATTGACGAT	EO53
PpSEC5c_RNAi_R_SalI	Cloning Sec5RNAi construct	AAAGTCGACGAAAGCTCCAGATGAGGATCCTC	EO54
PpSEC5d_RNAi_F_XhoI	Cloning Sec5RNAi construct	CACCCTCGAGGAAGACGACGAAGAAGAGGAGC	EO55
PpSEC5d_RNAi_R_SalI	Cloning Sec5RNAi construct	AAAGTCGACGTTTCTCGAAGCTCCCGCA	EO56
PpSec5a_qPCR_F	qRT-PCR Sec5RNAi lines	CGGTCAAACTTTCCGAGTACG	2772
PpSec5a_qPCR_R	qRT-PCR Sec5RNAi lines	TATGCCTCCATCACTGCCAAAT	2773
PpSec5b_qPCR_F	qRT-PCR Sec5RNAi lines	GGGGCAACGCCTATGTAGA	2768
PpSec5b_qPCR_R	qRT-PCR Sec5RNAi lines	ACACAGCCAGCTATTGCCTT	2769
PpSec5c_qPCR_F	qRT-PCR Sec5RNAi lines	ATGTCACAGGAGGAAATACAGG	EO164
PpSec5c_qPCR_R	qRT-PCR Sec5RNAi lines	GTTTGGAAGGTAGTCTGCAATGA	EO165
PpSec5d_qPCR_F	qRT-PCR Sec5RNAi lines	CTTCCGTGGCAACCCTCAAA	2770
PpSec5d_qPCR_R	qRT-PCR Sec5RNAi lines	CCCTGTGAGGCCTTCAATTCT	2771
PpEF1a_F	qRT-PCR Sec5RNAi lines	AATCATACATTTCACCTCGCC	2764
PpEF1a_R	qRT-PCR Sec5RNAi lines	GATCAGTGGGTAGAAGTGAC	2765

Table S3. Plasmids used in this study.

Plasmid	Usage	Source
pENTR-D-TOPO-AVR1	Cloning of AVR1 inducible expression vector	[15]
pENTR-D-TOPO-A-L	Cloning of AVR1 inducible expression vector	[15]
pENTR-D-TOPO-AVR1ΔT	Cloning of AVR1 inducible expression vector	[15]
pENTR-D-TOPO-myc-Cer-AVR1	Cloning of AVR1 inducible expression vector	This study
pENTR-D-TOPO-myc-Cer-AVR1ΔT	Cloning of AVR1 inducible expression vector	This study
pGWB20-AVR1-10myc	Cloning of AVR1 inducible expression vector	[15]
pGWB20-AVR1ΔT-10myc	Cloning of AVR1 inducible expression vector	This study
pENTR-D-TOPO-AVR1-10myc	Cloning of AVR1 inducible expression vector	This study
pENTR-D-TOPO-AVR1ΔT-10myc	Cloning of AVR1 inducible expression vector	This study
pPGX8	β-estradiol inducible expression	[30]
pPGX8-AVR1-myc	β-estradiol inducible AVR1 expression	This study
pPGX8-AVR1ΔT-myc	β-estradiol inducible AVR1ΔT expression	This study
pPGX8-myc-Cer-AVR1	β-estradiol inducible AVR1 expression	This study
pPGX8-myc-Cer-AVR1ΔT	β-estradiol inducible AVR1ΔT expression	This study
pENTR-D-TOPO-Sec5a	Cloning for yeast-two-hybrid assay	This study
pENTR-D-TOPO-Sec5b	Cloning for yeast-two-hybrid assay	This study
pENTR-D-TOPO-Sec5c	Cloning for yeast-two-hybrid assay	This study
pENTR-D-TOPO-Sec5d	Cloning for yeast-two-hybrid assay	This study
pDEST22-Sec5a	Yeast-two-hybrid assay	This study
pDEST22-Sec5b	Yeast-two-hybrid assay	This study
pDEST22-Sec5c	Yeast-two-hybrid assay	This study
pDEST22-Sec5d	Yeast-two-hybrid assay	This study
pDEST32-AVR1	Yeast-two-hybrid assay	[15]
pDEST32-AVR1ΔT	Yeast-two-hybrid assay	[15]
pDEST32-A-L	Yeast-two-hybrid assay	[15]
pDEST32-myc-Cer-AVR1	Yeast-two-hybrid assay	This study
pDEST32-myc-Cer-AVR1ΔT	Yeast-two-hybrid assay	This study
pGG626	Induced gene silencing in *P. patens*	[31]
pENTR-D-TOPO-Sec5RNAi	Gene silencing of all four *P. patens* homologs	This study
pGG626-Sec5RNAi	Gene silencing of all four *P. patens* homologs	This study

Table S4. *Physcomitrium patens* strains used in this study.

***P. patens* line**	**Representative clone(s)**	**Background strain**	**Reference**
AVR1-myc/Sec5b-GFP	66, 127, 212	Sec5b-GFP	This study
AVR1-myc/Sec5d-GFP	147, 201, 208	Sec5d-GFP	This study
AVR1ΔT-myc/Sec5b-GFP	5, 11, 224	Sec5b-GFP	This study
AVR1ΔT-myc/Sec5d-GFP	27, 126, 148	Sec5d-GFP	This study
myc-Cer-AVR1/Sec5b-GFP	119, 125, 174	Sec5b-GFP	This study
myc-Cer-AVR1/Sec5d-GFP	9, 11, 26	Sec5d-GFP	This study
myc-Cer-AVR1ΔT/Sec5b-GFP	27, 235, 422	Sec5b-GFP	This study
myc-Cer-AVR1ΔT/Sec5d-GFP	3, 15, 18	Sec5d-GFP	This study
Sec6RNAi	6	Sec6-GFP/Scamp4-mCherry	[28]
Sec5RNAi	18, 107	WT, Gransden isolate	This study
Sec5RNAi	58	mCherry-Tua1	This study
WT, Gransden isolate			[34]
Sec5b-GFP	42	WT, Gransden isolate	[34]
Sec5d-GFP	39	WT, Gransden isolate	[34]
mCherry-Tua1		WT, Gransden isolate	T. Miki, unpublished
