# Supplementary material for: Phytophthora infestans RXLR effector AVR1 disturbs the growth of Physcomitrium patens without affecting Sec5 localization
Source: PLoS One. 2021 Apr 8;16(4):e0249637. doi: 10.1371/journal.pone.0249637 (PMC8031463; doi:10.1371/journal.pone.0249637)
Supplement: S1 Raw images — (PDF) [file pone.0249637.s006.pdf]

Addendum to Figure S1 in Overdijk et al.

GENOTYPING

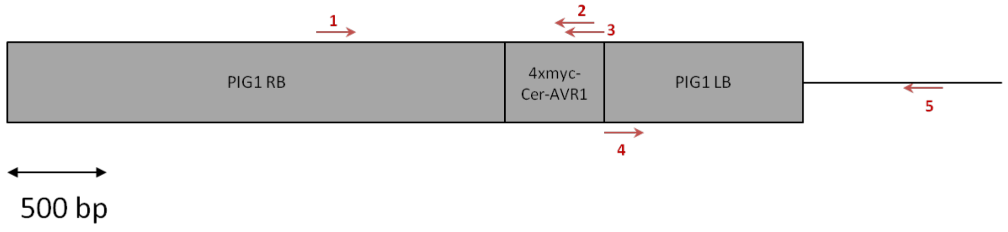

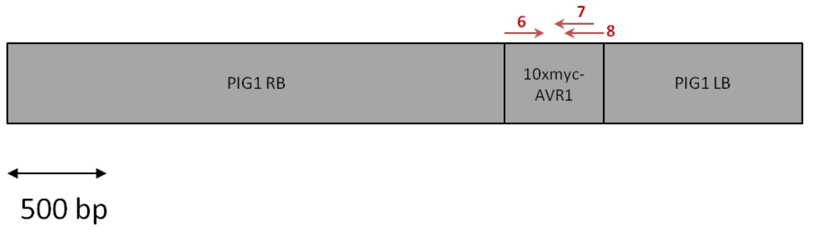

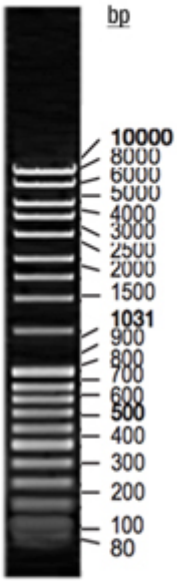

1: EO120
2: EO122
3: EO121
4: JK130
5: EO114
6: EO117
7: EO085
8: EO115

Primers and programs used in this study

Genotyping goal		Primers	Sequence (5'-3')	Length (min)	T.an (°C)	T.ext (°C)
AVR1_myc	F	EO117	CACCgcagaATGTTCGACCACG	00:02:00	55	72
	R	EO115	TTAAAATGGTACCACAACATGTCCACC			
AVR1dT_myc	F	EO117	CACCgcagaATGTTCGACCACG	00:01:30	55	72
	R	EO85	TTACCTCCACTTCACAGCCCGTGCC			
myc_Cer_AVR1	F	EO120	ATGGGGTTAATTAACGGTGAACAAAAGC	00:01:45	57	72
	R	EO121	AAATGGTACCACAACATGTCCACCAAG			
myc_Cer_AVR1dT	F	EO120	ATGGGGTTAATTAACGGTGAACAAAAGC	00:01:45	57	72
	R	EO122	CCTCCACTTCACAGCCCGTG			
Left Flank AVR1	F	JK130	TGCTAAGGCAGGGTTGGTTACG	00:04:00	53	67
	R	EO114	CCCTCTGCCGACCCATATTTC			

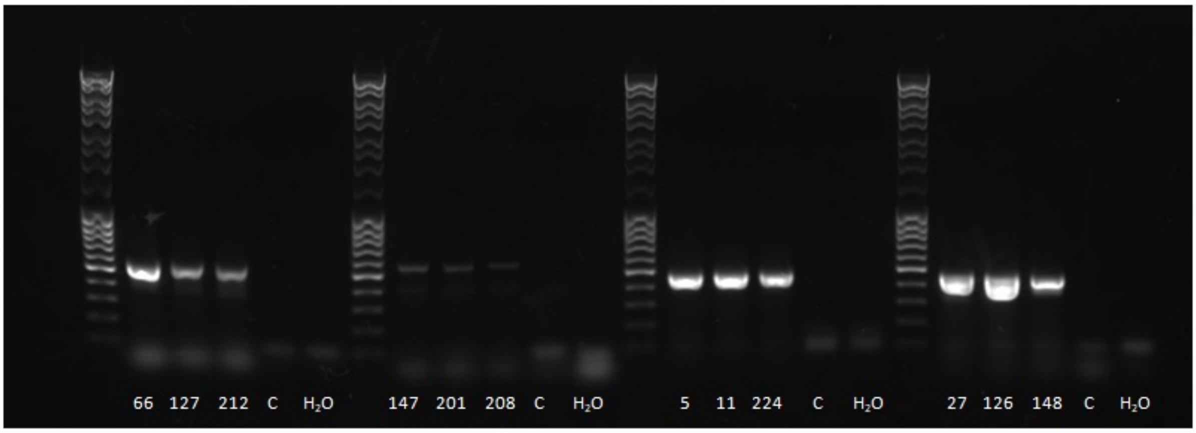

Sec5b_Amyc | Sec5d_Amyc | Sec5b_dTmyc | Sec5d_dTmyc

6 + 8: ±500bp | 6 + 8: ±500bp | 6 + 7: ±500bp | 6 + 7: ±500bp

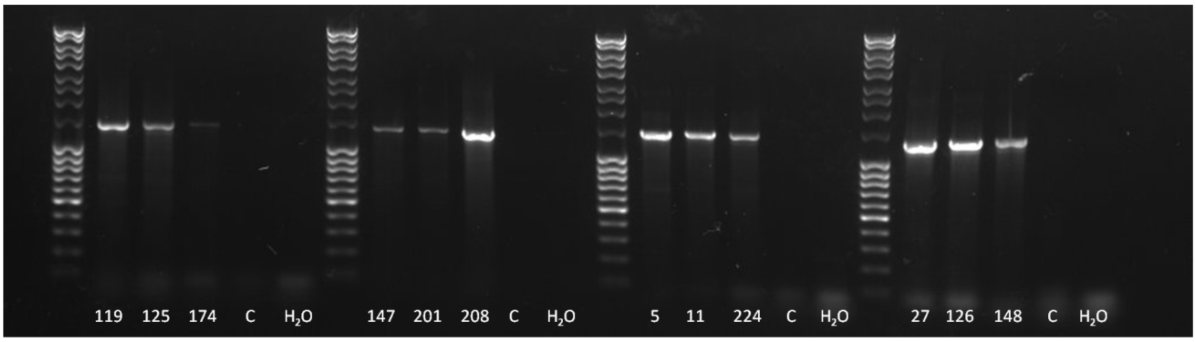

Sec5b_MCA | Sec5b_MCdT | Sec5d_MCA | Sec5d_MCdT

1 + 3: 1440bp | 1 + 2: 1337bp | 1 + 3: 1440bp | 1 + 3: 1337bp

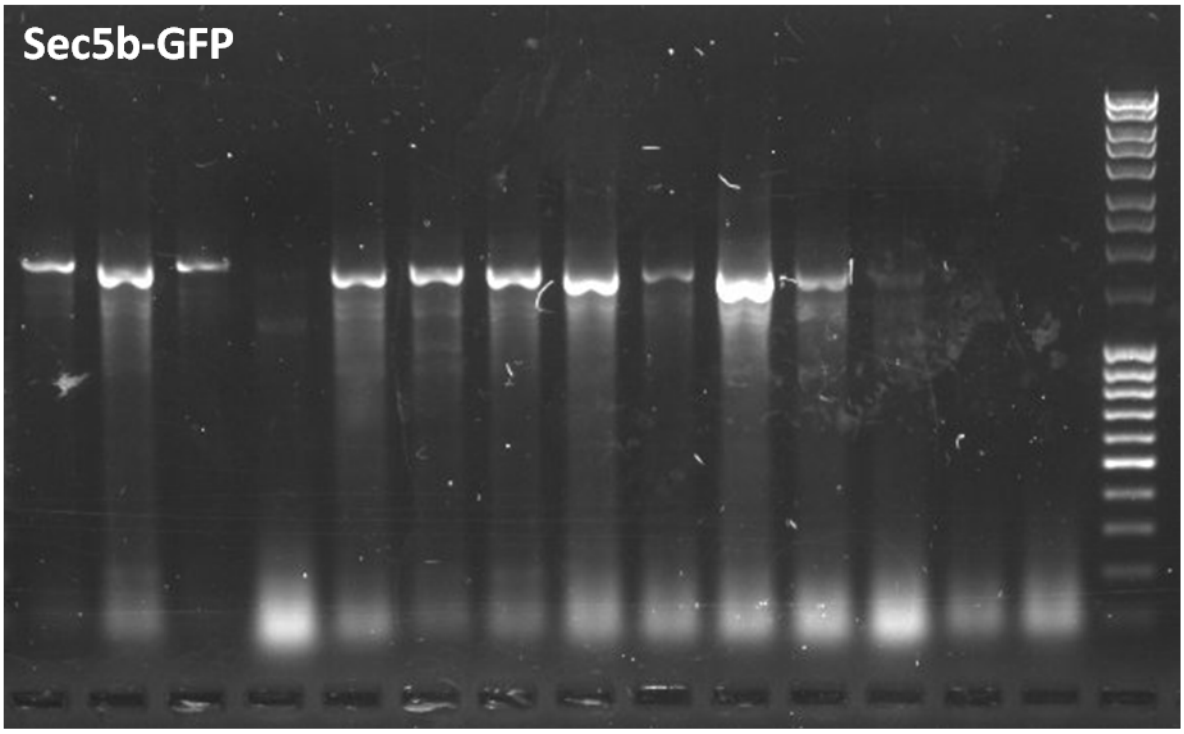

66 127 212 5 11 224 119 125 174 17 235 422 C H_2O

Amyc dTmyc MCA MCdT

4 + 5: ±1700kb

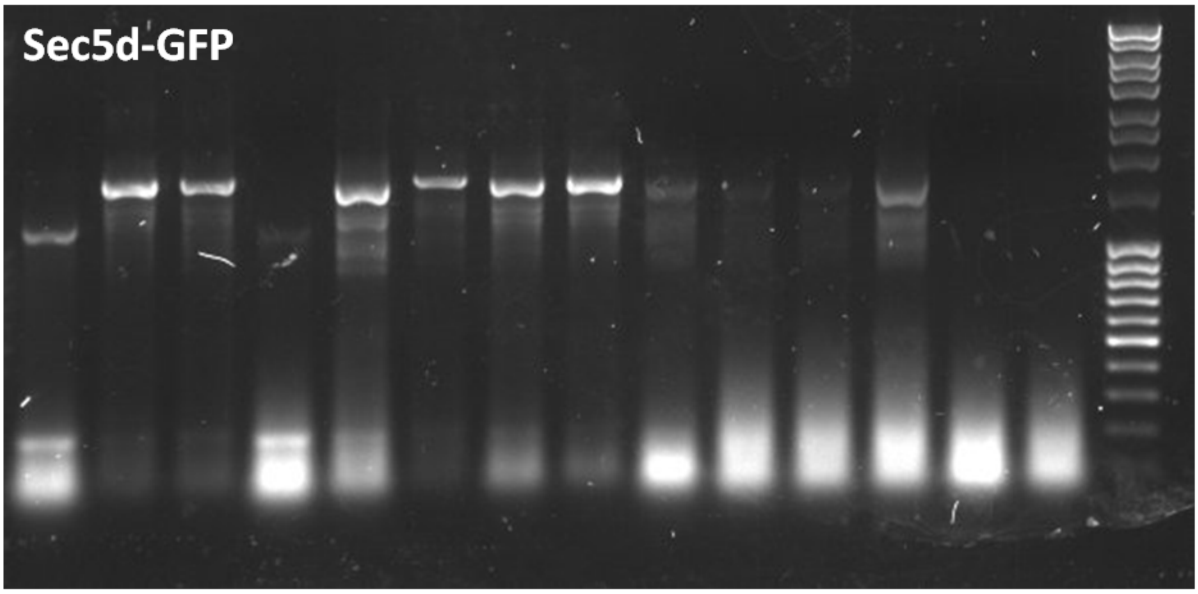

147 201 208 27 126 148 9 11 26 3 15 18 C H_2O

Amyc dTmyc MCA MCdT

4 + 5: ±1700kb
